# Multimodal Single-Cell Transcriptomic and Chromatin Accessibility Profiling Reveals Monocyte-Derived Macrophage Dynamics Following Ischemic Stroke

**DOI:** 10.3390/ijms27083657

**Published:** 2026-04-20

**Authors:** Milton H. Hamblin, Rabi Murad, Austin C. Boese, Huijie Huang, Rebecca A. Porritt, Tanvi Bobba, Jean-Pyo Lee

**Affiliations:** 1 Division of Biomedical Sciences, School of Medicine, University of California, Riverside, Riverside, CA 92521, USA; 2 NCI-Designated Cancer Center, Sanford Burnham Prebys Medical Discovery Institute, La Jolla, CA 92037, USA; rmurad@sbpdiscovery.org (R.M.); rporritt@sbpdiscovery.org (R.A.P.); 3 School of Medicine, Emory University, Atlanta, GA 30322, USA; austin.boese93@gmail.com; 4 Center for Neurological Diseases, Sanford Burnham Prebys Medical Discovery Institute, La Jolla, CA 92037, USA; hhuang@sbpdiscovery.org; 5 Health Sciences Center, Tulane University, New Orleans, LA 70112, USA; tbobba@tulane.edu

**Keywords:** ischemic stroke, monocyte-derived macrophages, single-cell transcriptomics, single-cell chromatin profiling

## Abstract

Ischemic stroke promotes monocyte recruitment to the injured brain and their differentiation into monocyte-derived macrophages (MDMs). These cells contribute to debris clearance but may also exacerbate neuroinflammation. However, the heterogeneity of MDM subsets and the phenotypic transitions that shape MDM functional states during the subacute phase of stroke remain incompletely characterized. To address this, we first performed single-cell RNA sequencing (scRNA-seq) to define the transcriptional landscape of the mouse brain 48 h after transient middle cerebral artery occlusion/reperfusion compared with sham controls. Reclustering of macrophage-lineage cells identified multiple MDM subsets, including a distinct *Cd68^hi^*/*Ctsd^hi^* MDM subset enriched for lysosomal and lipid-processing gene expression programs. Cell trajectory inference supported a transition from early recruited MDMs toward the *Cd68^hi^*/*Ctsd^hi^* state, accompanied by induction of transcriptomic networks that drive MDM function to favor a clearance-competent phenotype in response to ischemic stroke. Complementary single-cell ATAC sequencing (scATAC-seq) demonstrated cell type-specific chromatin remodeling after stroke and revealed MDM subclusters with accessibility at key loci regulating lysosomal function and lipid metabolism. Together, our findings define a cellular and regulatory framework of the subacute post-stroke brain and identify a lysosome-enriched *Cd68^hi^*/*Ctsd^hi^* MDM trajectory, highlighting endolysosomal and lipid-processing programs during early stroke recovery.

## 1. Introduction

Stroke remains a major cause of mortality and long-term disability in the United States [1]. Approximately 87% of strokes are ischemic in origin [1], most commonly arising from thrombotic occlusion of cerebral arteries. Although early restoration of perfusion is essential for tissue salvage, ischemia followed by reperfusion (IR) induces secondary injury characterized by blood–brain barrier (BBB) disruption and immune response amplification [2,3]. Brain damage, therefore, evolves beyond the acute ischemic event, with sustained immune activation contributing to subacute and chronic tissue loss [4,5]. Innate immune activation is a central feature of evolving IR injury. Resident microglia respond rapidly to ischemic stress, and within 24–48 h, circulating monocytes infiltrate the compromised BBB [6,7]. Experimental depletion and lineage-tracing studies demonstrate that infiltrating C-C chemokine receptor 2-positive (*Ccr2^+^)* monocytes give rise to monocyte-derived macrophages (MDMs) that are distinct from microglia and substantially contribute to early immune response amplification following ischemia [8,9,10]. Also, some MDM subsets upregulate antigen presentation machinery, including MHC class II complexes and co-stimulatory molecules, linking innate immune activation to adaptive immune responses [11,12,13].

Although MDMs can exacerbate neuroinflammation [4,6,7], they also contribute to debris clearance and tissue remodeling during recovery [14,15,16,17]. Efficient phagocytic clearance is linked to resolution of inflammation and improved functional recovery from stroke, whereas impaired clearance sustains immune signaling and worsens stroke outcomes [18]. Increasing evidence suggests that infiltrating MDMs transition along dynamic differentiation trajectories shaped by local injury-associated cues [19]. Effective removal of apoptotic and lipid-rich debris requires coordinated activation of lysosomal biogenesis, proteolytic machinery, membrane expansion, and lipid-handling pathways that enhance endolysosomal capacity [20,21,22,23]. Programs supporting V-ATPase–dependent acidification [23,24,25] and lipid processing [26] may define a degradative maturation state optimized for handling and clearing myelin debris [27] and apoptotic cells [28] within the post-ischemic niche. However, the transcriptional and epigenomic mechanisms governing this functional transition remain incompletely understood.

Advances in single-cell transcriptomics, specifically single-cell RNA-sequencing (scRNA-seq), enable high-resolution mapping of cellular heterogeneity within the ischemic brain [19,29,30,31,32], revealing transcriptional diversification within the myeloid compartment of stroke-afflicted brains. However, transcriptomic profiling alone does not reveal the epigenetic states underlying MDM transitions. Gene expression programs are established and maintained by the underlying chromatin landscape, including selectively accessible enhancers, promoters, and transcription factor binding sites [33,34,35]. Single-cell ATAC sequencing (scATAC-seq) reveals chromatin accessibility at single-cell resolution, enabling the determination of whether injury-associated MDM phenotypes reflect transient inflammatory activation or more durable epigenomic remodeling.

Here, we applied a multimodal single-cell strategy combining scRNA-seq and scATAC-seq to characterize MDM differentiation states in the subacute post-ischemic mouse brain. Using ipsilateral brain tissue harvested 48 h after transient middle cerebral artery occlusion/reperfusion (MCAO/R), we defined global cellular shifts and confirmed robust expansion of macrophage populations. Reclustering of macrophage lineage cells identified five distinct MDM subsets. Trajectory analysis revealed progressive differentiation toward two dominant MDM states: one enriched for antigen presentation programs and another characterized by lysosome-associated degradation and lipid processing pathways. We focused on the lysosome-enriched subset as a candidate reparative population emerging during the subacute phase. Early recruitment-associated MDMs displayed neuroinflammatory transcriptional signatures, whereas later-stage MDMs showed upregulation of genes involved in lysosomal biogenesis, membrane expansion, proteolytic machinery, V-ATPase assembly, and lipid metabolism. Complementary scATAC-seq profiling demonstrated that these transcriptionally defined MDM subsets were supported by distinct chromatin accessibility landscapes. This transcriptional and epigenomic progression suggests a functional transition toward a high-capacity degradative phenotype optimized for debris clearance. Collectively, these findings establish a regulatory framework for MDM maturation after stroke and identify a lysosome-enriched *Cd68^hi^*/*Ctsd^hi^* trajectory, defined by high expression of *Cd68* and *Ctsd* and reinforced by epigenomic activation of the acidification–proteolysis–lipid processing axis.

## 2. Results

### 2.1. Validation of Cerebral Infarction and Integrated Single-Cell Transcriptomic and Epigenomic Profiling

**Figure 1:**
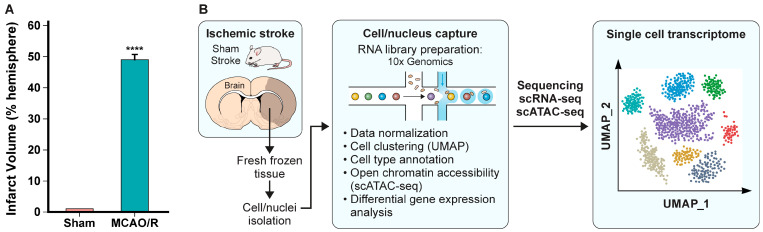
**Confirmation of ischemic injury and integrated single-cell transcriptomic and epigenomic profiling workflow.** (**A**) Ischemic injury was confirmed by a significant increase in infarct volume 48 h after MCAO/R. Data are presented as mean ± SEM (Sham, n = 8; MCAO/R, n = 6). The mean infarct volume in MCAO/R mice was 49.01 ± 1.38% of the ipsilateral hemisphere (**** *p* < 0.0001 vs. Sham). (**B**) Experimental workflow for integrated single-cell RNA sequencing (scRNA-seq) and single-cell ATAC sequencing (scATAC-seq). Ischemic brain tissue was dissociated into single-cell suspensions and processed using the 10× Genomics Chromium X platform. By integrating transcriptional and chromatin accessibility datasets from scRNA-seq and scATAC-seq, high-resolution cell clustering was performed through dimensionality reduction with UMAP.

**Figure A1:**
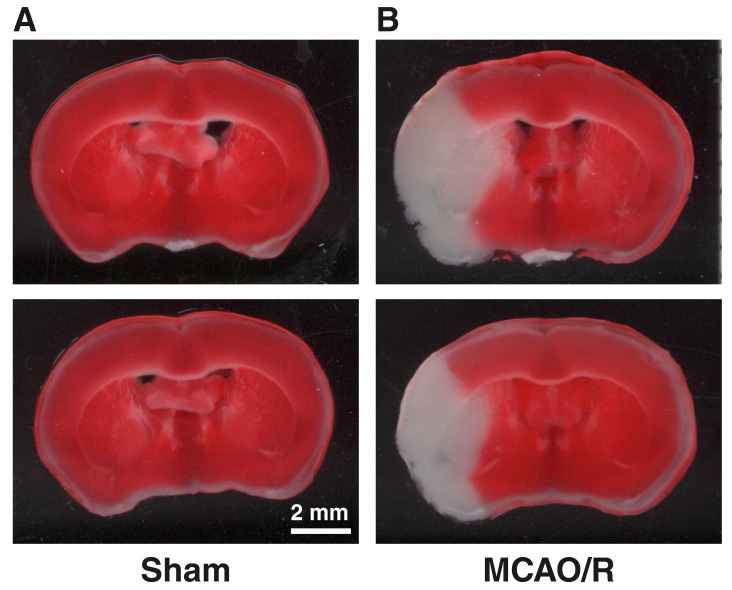
**TTC staining of brain tissue in the MCAO/R ischemic stroke mouse model.** TTC-stained mouse brain sections collected 48 h after surgery show infarcted tissue (white) and viable tissue (red). Ischemic damage is evident in the caudoputamen and cortex. Representative coronal sections are shown from mice that were (**A**) sham-operated and (**B**) subjected to MCAO/R. Each panel includes sections from two independent brains. MCAO/R, middle cerebral artery occlusion with reperfusion. Scale bar, 2 mm.

For the model of ischemic stroke, we used brains from young adult male mice 48 h after middle cerebral artery occlusion/reperfusion (MCAO/R). Brains from mice subjected to sham surgery served as the control. Ischemic injury was confirmed by a significant increase in infarct volume 48 h after MCAO/R, quantified by TTC staining (Figure A1A,B). Ischemic injury was localized to the ipsilateral MCA territory, involving cortical areas (sensorimotor cortex) and subcortical regions, primarily the caudoputamen (Figure A1B). Brains from MCAO/R mice had a mean infarct volume of 49.01 ± 1.38%, and brains from sham controls had negligible infarction (**** *p* < 0.0001) (Figure 1A and Figure A1A,B).

To assess transcriptomic and epigenomic changes in macrophages within the CNS 48 h after MCAO/R, we collected brains from the MCAO/R and sham control mice, generated single-cell suspensions and performed scRNA-seq and scATAC-seq. To identify cells with similar phenotypes, we performed Uniform Manifold Approximation and Projection (UMAP) analysis. We used known transcriptional signatures to identify the major populations of cells in the brain and then evaluated the macrophage subpopulations by transcriptional and epigenomic profiling (Figure 1B).

### 2.2. Single-Cell Transcriptomic Profiling Reveals Increased Macrophage Populations in Stroke Brains

**Figure 2:**
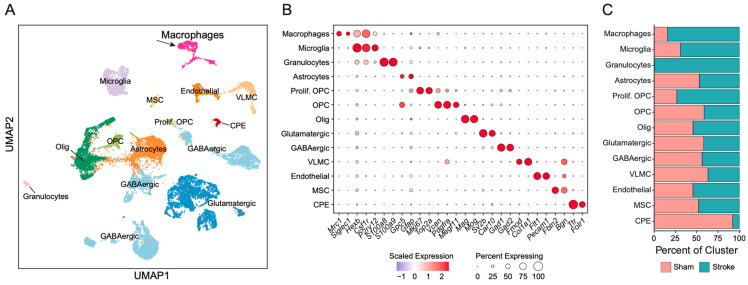
**Single-cell transcriptomic profiling reveals cellular heterogeneity and expansion of macrophage populations after ischemic stroke.** (**A**) UMAP visualization of single cells from sham (11,118 cells) and 48 h post-MCAO/R (8042 cells) mouse brains, colored by transcriptionally defined clusters. Major annotated populations include macrophages, microglia, granulocytes, astrocytes, oligodendrocytes (Olig), oligodendrocyte precursor cells (OPC), proliferating OPCs (Prolif. OPC), glutamatergic neurons, GABAergic neurons, endothelial cells, vascular and leptomeningeal cells (VLMC), mesenchymal stromal cells (MSC), and choroid plexus epithelial cells (CPE). (**B**) Dot plot of representative marker genes used for cluster annotation. Dot size indicates the proportion of cells expressing each gene within a cluster, and color intensity represents scaled average expression. (**C**) Stacked bar plots showing relative cell-type composition in sham and stroke samples, demonstrating a marked increase in macrophages following ischemic injury.

To define cellular heterogeneity following stroke, we performed scRNA-seq of stroke and sham brains. We obtained 19,160 high-quality cells after removal of low-quality and doublets and visualized the data using UMAP. Unsupervised clustering of the data from all of the cells from both sham and stroke brains identified distinct cell populations that were identified based on enrichment of lineage-specific transcripts (Figure 2A): macrophages, microglia, granulocytes, astrocytes, oligodendrocytes, oligodendrocyte precursor cells (OPCs), proliferating OPCs, glutamatergic neurons, GABAergic neurons, endothelial cells, vascular and leptomeningeal cells, mesenchymal stromal cells, and choroid plexus epithelial cells. Cell-type annotation was validated by targeted analysis of the expression of canonical marker genes (Figure 2B).

To quantify stroke-induced cellular shifts, we assessed each transcriptionally distinct cell population for the proportion of cells from the infarcted or sham brains comprising the population (Figure 2C). Stroke brains exhibited an increase in the proportion of proliferating OPCs and microglia. Stroke brains had a pronounced increase in the proportion of macrophages, and granulocytes were only found following stroke. These findings revealed substantial immune remodeling in the first 48 h in the post-ischemic brain. Although both macrophages and granulocytes increased following stroke, subsequent analyses focused on macrophages given their dynamic differentiation and functional plasticity during the subacute phase, in contrast to granulocytes, which are short-lived and exhibit limited transcriptional diversity.

### 2.3. Macrophage Clustering Identifies Distinct MDM Subsets and a Lysosome-Enriched Differentiation Trajectory After Stroke

**Figure 3:**
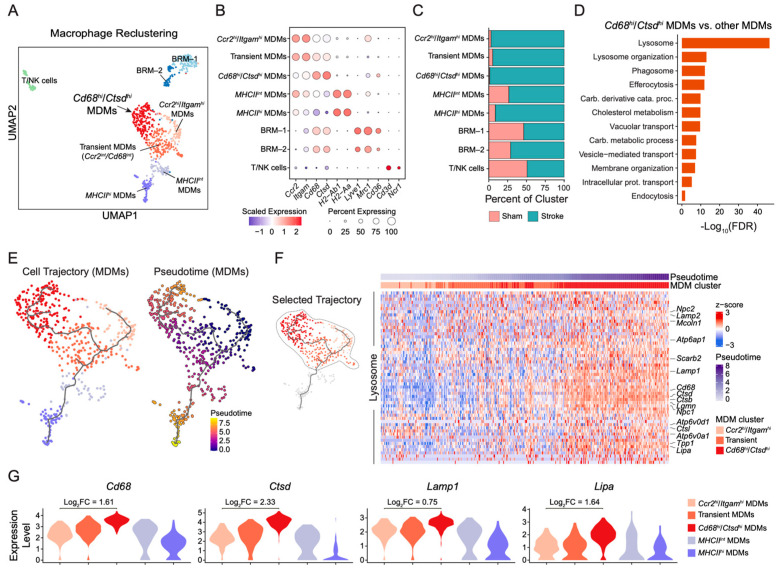
**Higher resolution reclustering of macrophages identifies distinct monocyte-derived macrophage (MDM) subsets and a lysosome-enriched differentiation trajectory following stroke.** (**A**) UMAP visualization of reclustered macrophage-lineage cells from sham and stroke samples. Distinct subclusters include *Ccr2^hi^*/*Itgam^hi^* MDMs, transient MDMs, *Cd68^hi^*/*Ctsd^hi^* MDMs, *MHCII^hi^* MDMs, *MHCII^int^* MDMs, brain-resident macrophages (BRM-1 and BRM-2), and a small T/NK cell population. (**B**) Dot plot of distinct marker genes defining each subcluster. Dot size represents the proportion of cells expressing each gene, and color intensity indicates scaled average expression. (**C**) Stacked bar plots showing the proportional distribution of macrophage subsets in sham and stroke, demonstrating expansion of monocyte-derived populations after injury. (**D**) Gene Ontology (GO) pathway enrichment analysis (using Metascape) of genes overexpressed in *Cd68^hi^*/*Ctsd^hi^* MDMs compared with other MDM subsets. Enriched pathways include lysosome organization, phagosome, vacuolar transport, vesicle-mediated transport, cholesterol metabolism, intracellular protein transport, membrane organization, and endocytosis. (**E**) Cell trajectory inference of MDM subclusters. Cells are colored by MDM subtype identity (**left**) and pseudotime (**right**) with *Ccr2^hi^*/*Itgam^hi^* MDMs selected as the root cluster for inferring pseudotime, revealing progression from *Ccr2^hi^*/*Itgam^hi^* and transient MDMs toward a *Cd68^hi^*/*Ctsd^hi^* state. (**F**) Heatmap of lysosome-associated trajectory genes plotted along *Ccr2^hi^*/*Itgam^hi^* to transient to *Cd68^hi^*/*Ctsd^hi^* MDM cell trajectory with increasing pseudotime. (**G**) Violin plots showing expression of representative lysosomal and lipid-processing genes (*Cd68*, *Ctsd*, *Lamp1*, *Lipa*) across MDM subsets. The y-axis represents normalized log-expression values generated using Seurat (LogNormalize), reflecting gene expression levels across all cells within each cluster. Log_2_FC values comparing *Ccr2^hi^*/*Itgam^hi^* and *Cd68^hi^*/*Ctsd^hi^* MDMs are provided for each gene.

To resolve macrophage heterogeneity following stroke, we clustered only the macrophage group of cells from both sham (161 cells) and stroke (616 cells) samples (Figure 3A). UMAP analysis identified five transcriptionally distinct subclusters of MDMs, two brain-resident macrophage clusters (BRM-1 and BRM-2), and a small T cell/Natural Killer (T/NK) cell population. The subgroups of MDMs included *Ccr2^hi^*/*Itgam^hi^* cells, *Cd68^hi^*/*Ctsd^hi^* cells, *MHCII^hi^* cells, *MHCII^int^* cells, and a subset that we categorized as “transient” MDMs. Cluster identities were confirmed by canonical marker gene expression (Figure 3B). *Ccr2* and *Itgam* defined infiltrating *Ccr2^hi^*/*Itgam^hi^* MDMs, *Cd68* and *Ctsd* defined the *Cd68^hi^*/*Ctsd^hi^* subset, *H2-Ab1* and *H2-Aa* defined *MHCII^hi^* and *MHCII^int^* populations, with the *MHCII^int^* also exhibiting intermediate *Ccr2* and *Itgam* expression, consistent with a phenotype intermediate between *Ccr2^hi^*/*Itgam^hi^* and *MHCII^hi^* MDMs. We defined transient MDMs as those with intermediate expression of *Ccr2* and *Cd68*, suggesting a phenotype intermediate between *Ccr2^hi^*/*Itgam^hi^* and *Cd68^hi^*/*Ctsd^hi^* MDMs. To determine how the macrophage populations changed following ischemic stroke, we assessed the proportion of each cell type (Figure 3C). BRM-1 and T/NK cells were equally distributed, with similar numbers from sham and stroke brains. The majority of cells within each MDM subpopulation originated from stroke brains. These changes in the MDM populations suggested recruitment and progressive differentiation of peripheral monocytes within 48 h after ischemic injury.

To define the functional characteristics of the *Cd68^hi^*/*Ctsd^hi^* MDM population, we performed differential gene expression analysis, which identified 593 genes enriched compared with other MDM clusters (FDR < 0.05, |log_2_FC| ≥ 0.25) (Table A1), followed by Gene Ontology (GO) enrichment analysis using this gene set (Figure 3D). Compared with the transcriptional profiles of the other MDM populations, *Cd68^hi^*/*Ctsd^hi^* MDMs were significantly enriched for lysosome-associated processes, such as lysosome organization, phagosomes, efferocytosis, membrane organization, vesicle-mediated transport, and endocytosis. Additionally, *Cd68^hi^*/*Ctsd^hi^* MDMs had a transcription profile enriched for cholesterol metabolism and carbohydrate metabolic processes. Collectively, this enrichment analysis indicated that *Cd68^hi^*/*Ctsd^hi^* MDMs are associated with phagocytosis and lysosome-mediated degradation.

The presence of MDM populations with marker profiles that were intermediate between *Ccr2^hi^*/*Itgam^hi^* cells and *Cd68^hi^*/*Ctsd^hi^* cells or *MHCII^hi^* cells suggested progression through transitional states. Trajectory inference using Monocle3 was therefore performed to model lineage relationships among MDM subsets. Pseudotime ordering indicated progression from *Ccr2^hi^*/*Itgam^hi^* to transient MDMs toward a *Cd68^hi^*/*Ctsd^hi^* state or toward an *MHCII^int^*-to-*MHCII^hi^* branch (Figure 3E). When *Ccr2^hi^*/*Itgam^hi^* MDMs were designated as the root population, *Cd68^hi^*/*Ctsd^hi^* and *MHCII^hi^* clusters occupied the highest pseudotime values, consistent with their positioning as terminal differentiation states. The trajectory toward the *Cd68^hi^*/*Ctsd^hi^* state was further examined to determine whether this transition was accompanied by induction of functionally linked gene modules.

**Figure A2:**
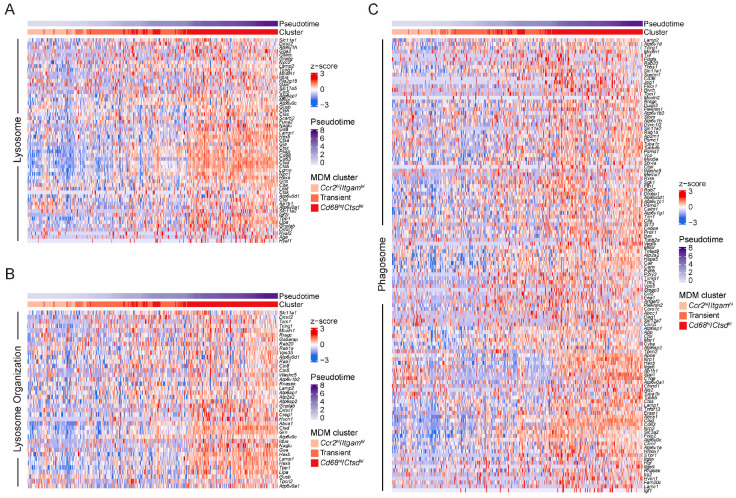
**Lysosome and phagosome gene expression patterns associated with functional heterogeneity of infiltrating MDM subsets after ischemic stroke.** Single-cell RNA-seq identified three infiltrating monocyte-derived macrophage (MDM) subsets: *Ccr2^hi^*/*Itgam^hi^ to transient to Cd68^hi^*/*Ctsd^hi^* MDMs arranged along the inferred pseudotime trajectory. Columns represent individual cells ordered by pseudotime progression, and rows denote genes associated with lysosomal function, lysosomal membrane organization, and phagosome pathways. The color scale indicates scaled gene expression (red, high; blue, low; z-score). (**A**) Heatmap (z-score) of lysosome-associated genes involved in biogenesis, proteolysis, and acidification (e.g., *Lamp1*, Cts family members, *Atp6v0d1*). (**B**) Heatmap (z-score) of genes regulating lysosomal membrane organization, vesicular trafficking, and V-ATPase assembly (e.g., *Cd68*, *Lamp1*, Atp6v0/Atp6v1 subunits, *Rab7*). (**C**) Heatmap (z-score) of phagosome-associated genes mediating engulfment, maturation, lysosome fusion, and degradative function (e.g., *Fcgr* family members, *Mertk*, *Cd68*, cathepsins, V-ATPase components). Across panels, *Cd68^hi^*/*Ctsd^hi^* MDMs exhibit elevated lysosome and phagosome gene expression relative to *Ccr2^hi^*/*Itgam^hi^* cells, with transient MDMs displaying intermediate profiles along the differentiation trajectory.

Cells from the *Ccr2^hi^*/*Itgam^hi^*, transient (*Ccr2^int^*/*Cd68^int^*), and *Cd68^hi^*/*Ctsd^hi^* clusters were ordered along increasing pseudotime (Figure 3F and Figure A2A–C). Differential expression analysis revealed progressive upregulation of lysosome-associated genes along this continuum. Heatmap visualization demonstrated gradual enrichment of transcripts encoding proteins involved in lysosomal biogenesis, acidification (V-ATPase subunits), proteolysis (cathepsins), and lipid processing as cells transitioned from early *Ccr2^hi^*/*Itgam^hi^* MDMs through the transient state toward the *Cd68^hi^*/*Ctsd^hi^* population (Figure 3F). Targeted analyses of genes enriched for lysosomes (Figure A2A), lysosome organization (Figure A2B), or phagosome (Figure A2C) showed *Cd68^hi^*/*Ctsd^hi^* MDMs exhibited an increase in the expression of these genes relative to *Ccr2^hi^*/*Itgam^hi^* cells, with transient MDMs displaying intermediate profiles along the differentiation trajectory, supporting the presence of a structured lysosome-centered maturation program. *Cd68^hi^*/*Ctsd^hi^* MDMs exhibited the highest expression of transcripts for cathepsins (*Ctsb*, *Ctsd*, *Ctsl*), lysosomal membrane proteins (*Lamp1*), V-ATPase subunits (*Atp6v0d1*, *Atp6v0a1*), and lipid-processing genes (*Lipa*), whereas *Ccr2^hi^*/*Itgam^hi^* MDMs displayed comparatively lower expression, and transient MDMs showed intermediate levels consistent with stepwise maturation. Along pseudotime, genes encoding V-ATPase subunits, lysosomal proteases, and lipid-processing proteins showed coordinated increases in expression, with the strongest enrichment observed in the *Cd68^hi^*/*Ctsd^hi^* state (Figure 3F). This coordinated progression supports the emergence of a metabolically specialized, lysosome-enriched MDM population within the first 48 h after IR injury. Analysis of selected lysosomal transcripts (*Lipa*, *Lamp1*, *Ctsd*) across MDM subgroups (Figure 3G) showed that these were the most abundant in *Cd68^hi^*/*Ctsd^hi^* cells and least abundant in *MHCII^hi^* cells, supporting divergence into distinct functional trajectories.

### 2.4. Single-Cell Chromatin Accessibility Profiling and Macrophage Clustering Reveal Chromatin Programs Associated with Lysosomal Activation and Lipid Handling

**Figure 4:**
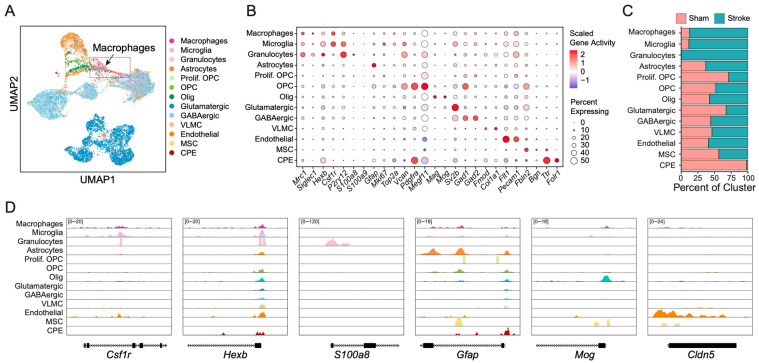
**Single-cell ATAC-seq defines cell type-specific chromatin accessibility landscapes in the subacute post-ischemic brain.** (**A**) UMAP visualization of scATAC-seq profiles from sham (6912 cells) and 48 h post-MCAO/R (9308 cells) brains, annotated using gene activity–based cell-type identities corresponding to those defined by scRNA-seq (Figure 2). The macrophage cluster is indicated (arrow). (**B**) Dot plot of inferred gene activity scores across annotated cell populations. Dot size represents the fraction of cells with detectable gene activity scores based on accessibility at promoter and gene body regions, and color intensity reflects scaled gene activity. (**C**) Relative cell-type composition in sham and stroke samples. Stroke brains exhibit increased representation of macrophages. (**D**) Genome browser tracks showing normalized chromatin accessibility at representative lineage-defining loci (*Csf1r*, *Hexb*, *S100a8*, *Gfap*, *Mog*, *Cldn5*) across major cell populations. Gene models are shown below each track.

One mechanism for cell differentiation is through epigenomic modifications that alter chromatin accessibility. To define the epigenomic changes in the post-ischemic brain, we performed scATAC-seq on ipsilateral brain tissue harvested 48 h after MCAO/R and from sham controls. UMAP analysis of 6912 cells from sham brains and 9308 cells from stroke brains was performed, and cell types were determined using gene activity–based identities corresponding to those defined by scRNA-seq clustering (Figure 4A). To align chromatin accessibility profiles with transcriptional identities, gene activity scores were computed based on chromatin accessibility at gene promoters and gene bodies and compared with corresponding gene expression patterns from scRNA-seq data. This integration enabled mapping of scATAC-seq clusters to scRNA-seq–defined cell identities. This analysis resolved distinct chromatin accessibility profiles corresponding to major neural, glial, vascular, and immune cell populations. Inferred gene activity analysis confirmed lineage-specific accessibility signatures across clusters (Figure 4B). Myeloid populations displayed enriched accessibility at canonical immune regulatory loci (such as *Csf1r*, *P2ry12*, and *Hexb*), whereas neuronal and glial populations retained accessibility at lineage-defining regulatory elements (*Sv2b*, *Gad1*, *Gad2* for neurons and *Gfap*, *Pdgfra*, and *Mog* for glia). The relative distribution of cell populations in sham and stroke brains was similar to that observed by scRNA-seq, with a marked expansion of myeloid populations following stroke (Figure 2C and Figure 4C). Specifically, chromatin accessibility profiling showed that macrophages, together with microglia and granulocytes, were enriched in the infarcted brain relative to sham (Figure 4C). To validate cluster annotation, we assessed chromatin accessibility at established lineage marker loci used for cell-type identification in Figure 4B. Genome browser tracks demonstrated cell-type–specific accessibility patterns (Figure 4D). Macrophages and microglia showed accessibility at *Csf1r* and *Hexb*, whereas granulocytes were the only population showing chromatin accessibility at *S100a8*. Astrocytes exhibited accessibility at *Gfap*, oligodendrocytes at *Mog* (a myelin-associated gene), and endothelial cells at *Cldn5*. These lineage-restricted accessibility profiles corroborate the gene activity–based annotations and confirm that chromatin landscapes faithfully reflect cell identity.

**Figure 5:**
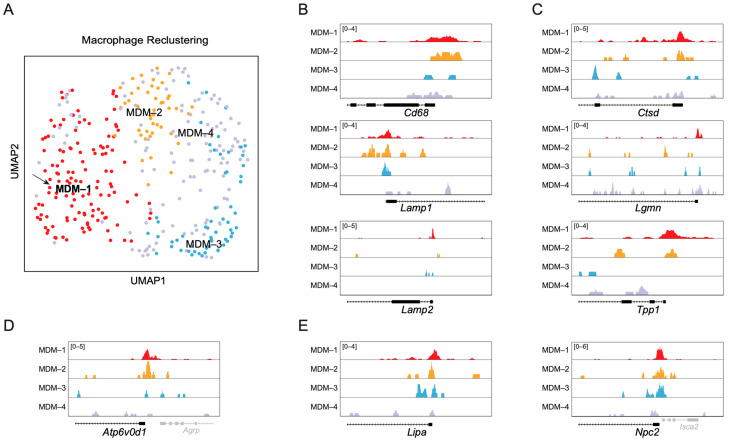
**Chromatin accessibility profiling defines lysosomal and lipid-processing regulatory programs in the *Cd68^hi^*/*Ctsd^hi^* MDM-1 subset.** (**A**) UMAP visualization of monocyte-derived macrophage subclusters (MDM-1 to MDM-4) defined by scATAC-seq. MDM-1 (arrow) corresponds to the *Cd68^hi^*/*Ctsd^hi^* population identified by scRNA-seq. (**B**–**E**) Genome browser tracks showing normalized chromatin accessibility at loci associated with lysosomal membrane expansion and phagolysosomal maturation (*Cd68*, *Lamp1*, *Lamp2*), lysosomal proteolysis (*Ctsd*, *Lgmn*, *Tpp1*), V-ATPase–dependent acidification (*Atp6v0d1*), and lipid hydrolysis/sterol trafficking (*Lipa*, *Npc2*) across MDM subclusters. Genome browser tracks display normalized signal intensity (range indicated per panel), with gene models shown below each locus. Increased accessibility at these loci in MDM-1 supports coordinated regulatory remodeling underlying the *Cd68^hi^*/*Ctsd^hi^* phagocytic state. Y-axis signal values represent normalized Tn5 insertion frequencies.

We next interrogated the epigenetic heterogeneity of these cells. UMAP-based reclustering of the macrophage population using chromatin accessibility profiles revealed four distinct subclusters of MDMs designated MDM-1 through MDM-4 (Figure 5A), indicating that post-ischemic MDMs generate discrete chromatin states. To determine whether these chromatin states correspond to functional specialization, we examined accessibility at lysosomal gene loci (Figure 5B–E). Genome browser tracks demonstrated that the MDM-1 subcluster displayed accessibility at canonical lysosomal loci, including *Cd68*, *Lamp1*, *Lamp2*, *Ctsd*, *Lgmn*, and *Tpp1*, supporting an epigenetically primed phagolysosomal program (Figure 5B,C) and indicating that these cells correspond to the *Cd68^hi^*/*Ctsd^hi^* population. MDM-2 showed a broadly similar accessibility pattern at these loci, particularly at *Cd68*, *Ctsd*, *Lamp1*, and *Atp6v0d1* (Figure 5B–D). The chromatin accessibility profiles of *Lipa* (encoding a lysosomal lipase) and *Npc2* (encoding a lysosomal cholesterol transporter) were similar among the MDM-1, MDM-2, and MDM-3 populations, whereas MDM-4 displayed a distinct accessibility pattern (Figure 5E), indicating that the MDM-4 population differentiated along a distinct pathway to a different state. These data suggested that MDM-3 and MDM-2 could be distinct populations on the differentiation path to MDM-1 (the *Cd68^hi^*/*Ctsd^hi^* population). Whether MDM-3 or MDM-2 represents the transient population identified by scRNA-seq or whether the transient population reflects both MDM-3 and MDM-2 is unclear.

Collectively, these findings demonstrate that MDM subsets are defined not only by transcriptional signatures but also by discrete chromatin accessibility programs. Enhanced accessibility at lysosomal and lipid-processing loci characterizes the *Cd68^hi^*/*Ctsd^hi^* phagocytic state, whereas alternative accessibility configurations distinguish other MDM populations, supporting an epigenetic basis for divergent differentiation trajectories.

## 3. Discussion

In this study, we combined single-cell transcriptomic and epigenomic profiling to define MDM subpopulations, differentiation states, and gene expression programs during the subacute phase of ischemic stroke. At 48 h after MCAO/R, scRNA-seq revealed cellular changes consistent with early immune activation, including marked expansion of infiltrating MDM populations when compared to brains from sham controls, which is consistent with established features of early stroke pathology [4,6,7] and prior single-cell studies of the injured brain in aged animals [36]. Here, we provide a detailed characterization of MDM subpopulations and their differentiation states in the subacute stroke phase, filling a gap in understanding early IR responses.

Trajectory analysis positioned *Ccr2^hi^*/*Itgam^hi^* MDMs, transient states, and *Cd68^hi^*/*Ctsd^hi^* MDMs along one differentiation continuum, whereas *MHCII^int^* and *MHCII^hi^* subsets followed a distinct branch from the *Ccr2^hi^*/*Itgam^hi^* MDMs. These findings align with prior studies demonstrating progressive maturation of infiltrating *Ccr2^+^* monocytes into specialized macrophage states in injured CNS tissue [8]. While the *Cd68^hi^*/*Ctsd^hi^* MDM population is characterized by enrichment of lysosomal and lipid-processing programs based on integrated transcriptomic and chromatin accessibility analyses, these findings are consistent with a degradative, debris-clearing state. Additional functional validation (e.g., phagocytic or lysosomal activity assays) would further substantiate this interpretation and would be important to address in future studies. Consistent with this interpretation, Yu et al. previously reported that border-associated macrophages undergo dynamic transcriptional changes after stroke and participate in immune pathway regulation [37]. *MHCII^hi^* macrophages are associated with antigen-presenting and immune-activating features [38], while *Cd68* is widely used as a macrophage marker [39]. Increased *Ctsd* expression is indicative of enhanced lysosomal proteolytic capacity [40], a feature commonly linked to metabolically adaptive macrophage programs [41]. Early recruited *Ccr2^hi^*/*Itgam^hi^* MDMs are associated with cytokine signaling and leukocyte recruitment, whereas the *Cd68^hi^*/*Ctsd^hi^* population maps to later pseudotime and exhibits coordinated transcriptomic and epigenetic programs consistent with lysosomal proteolysis, membrane expansion, acidification, and lipid handling.

Following CNS infiltration, MDMs contribute to early post-stroke immune responses through cytokine production, leukocyte recruitment, and antigen presentation [4,6,7]. Lineage-tracing studies confirm that infiltrating *Ccr2^+^* monocytes amplify early immune responses after ischemic stroke [8,9,32]. MDMs upregulate MHC class II, bridging innate and adaptive immunity [11,12,13] and shaping post-stroke T cell responses [5,42]. Also, MDMs contribute to debris clearance and tissue remodeling, thus limiting secondary injury [43,44]. Effective debris clearance requires coordinated phagocytosis and lysosome-dependent degradation. Following engulfment of apoptotic neurons and myelin fragments, phagosomes mature through sequential acquisition of endosomal and lysosomal components. Fusion with lysosomes enables degradation of proteins and lipids by cathepsins and acidic hydrolases that depend on lysosomal acidification through the vacuolar H^+^-ATPase (V-ATPase) complex [45].

Lysosomal function is also dependent on cholesterol and lipid processing. Lysosomal acid lipase (LIPA) hydrolyzes cholesteryl esters and triglycerides within lysosomes, while cholesterol export depends on coordinated NPC2–NPC1 transfer [46]. Disruption of cholesterol clearance impairs myeloid function and can exacerbate CNS pathology [47,48,49]. Failure of this lysosome–cholesterol axis promotes intracellular lipid accumulation and lipid-laden myeloid states that sustain immune signaling and alter tissue homeostasis. These findings position sterol flux as a contributor to the metabolic landscape influencing immune dynamics after ischemia. Since myelin debris is highly enriched in cholesterol and sphingolipids [50,51], efficient lysosomal processing is likely critical for limiting secondary immune response amplification after stroke. Furthermore, lysosomes function not merely as degradative organelles but also as metabolic and signaling hubs that integrate nutrient availability with mTORC1 activity, transcriptional regulation of lysosomal biogenesis, and cellular metabolic reprogramming. The TREM2–APOE signaling axis further links lipid sensing to macrophage state transitions [52]. These observations collectively suggest that acquisition of robust lysosomal acidification and cholesterol processing capacity may define a macrophage state associated with enhanced lysosomal and lipid-processing capacity. A central mechanistic insight from our trajectory analysis is increased activation of coordinated lysosomal modules: proteolytic machinery (*Ctsd*, *Lgmn*, *Tpp1*, *Ctsl*, *Ctsb*), lysosomal membrane expansion (*Lamp1*, *Lamp2*, *Cd68*, *Cd63*), V-ATPase–mediated acidification (*Atp6v0*/*Atp6v1* subunits, *Atp6ap1*, *Dmxl1*), and lipid-processing pathways (*Lipa*, *Npc2*, *Trem2*, *Apoe*, *Lgals3*).

A major advance of this study is the combination of scRNA-seq and scATAC-seq to connect macrophage gene expression signatures with chromatin states. Tissue macrophage identity is shaped by local enhancer landscapes responsive to environmental signals and immune-related stimuli [33,34,35]. In our scATAC-seq dataset, MDM subclustering revealed distinct chromatin accessibility states, with the *Cd68^hi^*/*Ctsd^hi^*-equivalent subset showing enriched accessibility at loci governing lysosomal membrane expansion (*Lamp1*, *Lamp2*, *Cd68*), proteolysis (*Ctsd*, *Lgmn*, *Tpp1*), acidification (*Atp6v0d1*), and lipid handling (*Lipa*, *Npc2).* These data indicate that the *Cd68^hi^*/*Ctsd^hi^* MDM state is stable and epigenetically reinforced. Our analysis primarily focused on promoter-associated accessibility. While intronic peaks observed in the scATAC-seq data may represent putative regulatory elements (e.g., enhancers), we did not formally annotate or functionally validate these regions, and, therefore, cannot determine whether they regulate the host gene or distal targets. In addition, chromatin accessibility does not uniformly correlate with gene expression. For instance, although decreased *Ccr2* expression in *CD68^hi^*/*Itgam^hi^* MDMs may be associated with reduced accessibility at regulatory regions, this relationship may not be consistent across all loci and likely reflects cell state–dependent regulatory dynamics.

Our findings refine prevailing models of post-stroke immune activation [4,5] by revealing functional specialization within infiltrating MDMs. Early recruited *Ccr2^hi^*/*Itgam^hi^* MDMs are associated with cytokine signaling and neurovascular changes, whereas the later appearing *Cd68^hi^*/*Ctsd^hi^* state is consistent with specialization toward lysosomal degradation and lipid metabolism. This population and the mechanisms regulating it warrant further investigation. Efficient processing of myelin fragments and cellular debris is critical for limiting secondary tissue damage and preventing prolonged immune activation [43]. Thus, the lysosome–lipid axis identified here may represent a key determinant of macrophage functional outcomes during the early phase of stroke.

While our data define a lysosome- and lipid-processing MDM state during early stroke, this study examines a single subacute time point (48 h post-MCAO/R), corresponding to a period of active innate immune activation. Although this time point is near the peak of MDM infiltration, it likely contains a mixture of recently infiltrating monocytes as well as more differentiated MDM states. Consistent with this heterogeneity, our trajectory/pseudotime analysis suggests a continuum of transcriptional states within the MDM population. Macrophage phenotypes are highly dynamic, and the transcriptional program identified here likely represents one stage within a broader continuum of MDM differentiation. However, additional temporal sampling (e.g., 24 h, 72 h, or later stages after stroke) would provide stronger temporal resolution and further strengthen the biological interpretation of these inferred differentiation dynamics. Future spatiotemporal studies incorporating multiple time points and spatial transcriptomic and proteomic approaches will help clarify how these MDM states evolve across anatomical niches and whether they persist, resolve, or contribute to chronic immune dysregulation.

In summary, our multimodal single-cell transcriptomic and epigenomic analyses define a regulatory framework for MDM differentiation during subacute ischemic stroke, wherein early MDMs transition toward a *Cd68^hi^*/*Ctsd^hi^* lysosome- and lipid-processing phenotype. This specialization is reinforced by chromatin accessibility remodeling at corresponding loci, indicating durable epigenomic reprogramming. These findings position lysosomal acidification and lipid metabolism as central mechanisms governing MDM state transitions and provide a high-resolution framework for targeting clearance programs to modulate immune responses and improve early stroke repair.

## 4. Materials and Methods

### 4.1. Animals

C57BL/6J male mice (8–12 weeks of age) were obtained from The Jackson Laboratory (Bar Harbor, ME, USA). Mice were housed in a temperature-controlled facility (18–22 °C) under a 12 h light/12 h dark cycle with ad libitum access to standard chow and water. 

### 4.2. Animal Model of Stroke

All experimental procedures involving animals were reviewed and approved by the Institutional Animal Care and Use Committees (IACUC) of Tulane University (New Orleans, LA, USA) and the University of California, Riverside (Riverside, CA, USA). All animal studies were conducted in accordance with institutional guidelines, the American Veterinary Medical Association recommendations, and the National Institutes of Health Guide for the Care and Use of Laboratory Animals. This study adhered to the ARRIVE 2.0 guidelines.

Transient focal cerebral ischemia was induced using a well-established intraluminal filament model based on the Longa method [53]. Surgical procedures were performed as previously described [54,55,56,57,58]. Mice were anesthetized with isoflurane (induction 3–4%, maintenance 1–2% in oxygen) during the surgical procedure. Briefly, a 6-0 nylon monofilament (Doccol Corporation, Sharon, MA, USA) was advanced to occlude the middle cerebral artery (MCA) for 1 h, followed by withdrawal to allow reperfusion. Sham-operated controls underwent filament insertion without sustained occlusion. Regional cerebral blood flow (rCBF) was monitored using transcranial laser Doppler flowmetry (Perimed AB, Järfälla, Sweden) to verify occlusion and reperfusion. A reduction in rCBF to less than 20% of baseline (≥80% reduction) confirmed successful MCA occlusion, and restoration to more than 90% of baseline levels indicated effective reperfusion. Brain tissue used for downstream analyses excluded the olfactory bulbs and cerebellum.

### 4.3. Quantification of Infarct Volume

Ischemic injury was assessed by triphenyl tetrazolium chloride (TTC, Sigma-Aldrich, St. Louis, MO, USA) staining of coronal brain sections. At 48 h following MCAO/R, brains were sectioned into 1 mm slices and incubated in 2% TTC solution as previously published [54,55,56,57,58]. Infarcted regions were quantified using ImageJ software (version 1.54, National Institutes of Health, Bethesda, MD, USA). To account for cerebral edema, infarct volume was calculated as a percentage of the contralateral hemisphere using the following formula: [(contralateral hemisphere volume − (ipsilesional hemisphere volume − infarct volume))/contralateral hemisphere volume] × 100. Although TTC staining is widely used to assess infarct volume in experimental stroke models, it may overestimate lesion size compared to MRI or histological approaches.

### 4.4. scATAC-seq Library Preparation

Flash-frozen sham brains (n = 4) and ipsilateral hemispheres of stroke brains (n = 4) were pooled within each condition prior to processing in Miltenyi nuclei extraction buffer containing 0.2 U/μL RNase inhibitor using a gentleMACS Octo Dissociator (Program 4C_nuclei_1, Miltenyi Biotec, Bergisch Gladbach, Germany) according to the manufacturer’s protocol. Miltenyi Debris Removal Solution was applied to the nuclei suspension. The mixture was centrifuged at 1500× *g* for 5 min, and the pelleted nuclei were resuspended in 500 μL resuspension buffer. Nuclei were counted, and scATAC-seq was performed with the Next GEM Single Cell ATAC Kit v2 (10× Genomics, Pleasanton, CA, USA) according to the user guide (CG000496, Rev B). Briefly, nuclei were transposed with Tn5 transposase to fragment accessible chromatin and insert adapters, then partitioned into Gel Beads-in-Emulsion (GEMs) using the 10× Genomics Chromium X system (10× Genomics), and DNA fragments were barcoded with cell-specific barcodes. After GEM recovery, barcoded fragments were PCR amplified and converted into indexed sequencing libraries. Libraries were sequenced to a depth of 2 billion paired-end (PE) reads with an Element Biosciences AVITI Sequencing platform using the AVITI 2 × 75 High Output Cloudbreak Freestyle Kit (Element Biosciences, San Diego, CA, USA). 

### 4.5. scRNA-seq Library Preparation

Fixed single-cell suspensions were prepared from pooled flash-frozen sham control brains (n = 4) and pooled ipsilateral hemispheres of stroke brains (n = 4) using the Chromium Next GEM Single Cell Fixed RNA Preparation kit (10× Genomics, Pleasanton, CA, USA) according to the user guide (CG000553, Rev B). Cell dissociation was performed using the gentleMACS Octo Dissociator (Miltenyi Biotec, Auburn, CA, USA). Libraries were constructed using the single-plex Chromium Fixed RNA Profiling kit (CG000477, Rev C, 10× Genomics). Briefly, whole-transcriptome probe pairs were hybridized to fixed cells during a 20 h incubation at 42 °C, followed by washing to remove excess probes. Cells were then partitioned into Gel Beads-in-Emulsion (GEMs) using the Chromium X system (10× Genomics). Hybridized probes were ligated and extended to generate barcoded molecules containing probe barcodes, cell-specific barcodes, and unique molecular identifiers (UMIs). Libraries were subsequently amplified and indexed to generate sequencing-ready libraries. Libraries were sequenced to a depth of 2 billion paired-end (PE) reads with an Element Biosciences AVITI Sequencing platform using the AVITI 2 × 75 High Output Cloudbreak Freestyle Kit (Element Biosciences).

### 4.6. scRNA-seq Data Processing and Analysis

Raw scRNA-seq data were processed using Cell Ranger multi v8.0.0 (https://www.10xgenomics.com/support/software/cell-ranger/latest, accessed 1 July 2024), mouse mm10 reference genome (refdata-gex-mm10-2020-A), and mouse transcriptome probe set v1.0 (mm10-2020-A). Cell Ranger outputs for sham and stroke samples were aggregated and normalized for sequencing depth using cellranger aggr with the parameter --normalize=mapped. Mean number of reads per cell was 16,504, median number of UMI counts per cell was 4587, and median number of genes detected per cell was 2516. Doublets for each sample were identified using scds v1.24.0 [59] in R v4.5.1. Following removal of low-quality cells and doublets, we obtained 11,118 and 8042 cells for sham and stroke samples, respectively. To account for differences in total cell numbers between conditions, cell-type proportions were normalized per 10,000 cells.

Integration and downstream analyses of the scRNA-seq samples were performed using Seurat v5.3.0 [60] in R v4.5.1. The aggregated Cell Ranger output count matrix was converted to a Seurat object using CreateSeuratObject, retaining cells with ≥200 detected genes and genes expressed in at least 5 cells. Doublets identified using scds analysis and low-quality cells with high mitochondrial content (>10%) were excluded from further downstream analyses. Raw counts were normalized using the NormalizeData function. The top 2000 highly variable genes were then identified using FindVariableFeatures. Data were scaled using ScaleData (regressing out nCount_RNA and mitochondrial percentage), followed by principal component analysis (RunPCA) using highly variable genes. Samples were integrated using the IntegrateLayers function with parameters method = “HarmonyIntegration” and orig.reduction = “pca”. Cell clustering was performed using RunUMAP, FindNeighbors, and FindClusters with resolution = 0.5 and principal components accounting for 90% cumulative variance. Cluster marker genes were determined using FindAllMarkers with parameters only.pos = TRUE, and min.pct = 0.25.

Macrophages were reclustered using SCTransform in Seurat. The macrophage cluster was subsetted, and the resulting Seurat object was slimmed using DietSeurat with assays = “RNA”. SCTransform was performed with parameters variable.features.n = 1300 and vars.to.regress = c(“nCount_RNA”, “percent.mt”). PCA was performed using RunPCA, and samples were integrated using IntegrateLayers with parameters orig.reduction = “pca”, method = “HarmonyIntegration”, and assay = “SCT”. Cell clustering was performed using RunUMAP, FindNeighbors, and FindClusters with resolution = 0.5 using the top 20 principal components.

Cell trajectory and pseudotime analysis were performed using Monocle3 [61] v1.4.26 in R v4.5.1. Cell trajectory analysis was performed using learn_graph and order_cells functions with the *Ccr2^hi^*/*Itgam^hi^* MDMs cluster as the root node. Pseudotime values were calculated using the pseudotime function. Plots were generated using Seurat, Monocle3, and ggplot2 v4.0.2 in R v.4.5.1.

### 4.7. scATAC-seq Data Processing and Analysis

Raw scATAC-seq data were processed using Cell Ranger ATAC v2.1.0 (patched version obtained from 10× Genomics Support to process AVITI sequenced data, accessed March 2024, support@10xgenomics.com) and the mouse mm10 reference genome (refdata-cellranger-arc-mm10-2020-A-2.0.0).

Downstream analysis of scATAC-seq data was performed using Signac v1.15.0 and Seurat v5.3.0 in R v4.5.1. For each sample, Cell Ranger ATAC output was converted to ChromatinAssay, followed by Seurat object using *CreateChromatinAssay* and *CreateSeuratObject*, respectively. Gene annotations were obtained from EnsDb.Mmusculus.v79 and added to Seurat objects. QC metrics were computed using *NucleosomeSignal* and *TSSEnrichment*. High-quality cells meeting the following criteria were retained: peak_region_fragments > 1000, peak_region_fragments < 100,000, pct_reads_in_peaks > 20, blacklist_ratio < 0.025, nucleosome_signal < 4, and TSS.enrichment > 2. The sham and stroke samples were merged using a unified set of peaks identified using the *reduce* function. Merged data were normalized using *RunTFIDF*. Top features were identified using *FindTopFeatures* with parameter “min.cutoff = 20”. Dimension reduction was performed using RunSVD (singular value decomposition). Cell clustering was performed using *RunUMAP* with parameters “dims = 2:10, reduction = ‘lsi’”, *FindNeighbors* with parameters “reduction = ‘lsi’, dims = 2:10”, and *FindClusters* with parameters “algorithm = 3, resolution = 1.2”. Gene activity levels were computed by quantifying chromatin accessibility associated with each gene using the *GeneActivity* function. Gene activity levels were normalized using NormalizeData with parameters “assay = ‘RNA’, normalization.method = ‘LogNormalize’, scale.factor = median(nCount_RNA)”. Cluster marker genes were identified using *FindAllMarkers* on gene activity levels. Annotations of clusters using scRNA-seq were performed with label transfer using *FindTransferAnchors* and *TransferData.*

### 4.8. Statistical Analysis

Statistical analyses were performed using GraphPad Prism 10 and SPSS 29. For comparisons between two independent groups, a two-tailed unpaired Student’s *t*-test was used. To determine dissimilarities between multiple groups, one-way ANOVA followed by Fisher’s LSD post hoc test was performed. *p* < 0.05 was considered statistically significant. Data are presented as mean ± SEM.

## Data Availability

Raw and processed scRNA-seq and scATAC-seq data are deposited in GEO under accession IDs GSE326241 and GSE326118, respectively.

## Abbreviations

*Apoe*	Apolipoprotein E
ARRIVE	Animal Research Reporting of In Vivo Experiments
*Atp6ap1*	ATPase H+ transporting accessory protein 1
*Atp6v0a1*	ATPase H+ transporting V0 subunit a1
*Atp6v0d1*	ATPase H+ transporting V0 subunit d1
BBB	Blood–brain barrier
BRM	Brain-resident macrophage
*Ccr2^hi^*	C-C motif chemokine receptor 2, high expression
*Ccr2^int^*	C-C motif chemokine receptor 2, intermediate expression
*Cd63*	Cluster of Differentiation 63
*Cd68*	Cluster of Differentiation 68
*Cldn5*	Claudin 5
CNS	Central nervous system
CPE	Choroid plexus epithelial cells
*Csf1r*	Colony-stimulating factor 1 receptor
*Ctsb*	Cathepsin B
*Ctsd*	Cathepsin D
*Ctsl*	Cathepsin L
*Dmxl1*	Dmx like 1
*Fcgr*	Fc gamma receptor (general designation for family members)
*Gad1*	Glutamate decarboxylase 1
*Gad2*	Glutamate decarboxylase 2
*Gfap*	Glial fibrillary acidic protein
GO	Gene Ontology
*H2-Aa*	Histocompatibility 2, class II antigen A, alpha
*H2-Ab1*	Histocompatibility 2, class II antigen A, beta 1
*Hexb*	Hexosaminidase subunit beta
HT	Hemorrhagic transformation
IACUC	Institutional Animal Care and Use Committee
IR	Ischemia/reperfusion
*Itgam*	Integrin subunit alpha M
*Lamp1*	Lysosomal-associated membrane protein 1
*Lamp2*	Lysosomal-associated membrane protein 2
*Lgals3*	Galectin 3
*Lgmn*	Legumain
*Lipa*	Lipase A, lysosomal acid type
LIPA	Lysosomal acid lipase
MCAO/R	Middle cerebral artery occlusion/reperfusion
MDM	Monocyte-derived macrophage
*Mertk*	MER proto-oncogene, tyrosine kinase
MHCII	Major histocompatibility complex class II
*Mog*	Myelin oligodendrocyte glycoprotein
MSC	Mesenchymal stromal cells
NK	Natural killer cells
*Npc2*	Niemann-Pick type C2 protein
Olig	Oligodendrocytes
OPC	Oligodendrocyte precursor cell
*P2ry12*	Purinergic receptor P2Y12
PCA	Principal Component Analysis
PCR	Polymerase chain reaction
*Pdgfra*	Platelet-derived growth factor receptor alpha
PE	Paired Ends
*Rab7*	RAB7, member of the RAS oncogene family
rCBF	Regional cerebral blood flow
*S100a8*	S100 calcium-binding protein A8
scATAC-seq	Single-cell Assay for Transposase-Accessible Chromatin sequencing
scRNA-seq	Single-cell RNA sequencing
SEM	Standard error of the mean
*Sv2b*	Synaptic vesicle glycoprotein 2b
T/NK	T cells and natural killer cells
*Tpp1*	Tripeptidyl peptidase 1
*Trem2*	Triggering receptor expressed on myeloid cells 2
TTC	2,3,5-triphenyltetrazolium chloride
UMAP	Uniform Manifold Approximation and Projection
V-ATPase	Vacuolar H+-ATPase
VLMC	Vascular and leptomeningeal cells

## Appendix A

**Table A1:** Differential gene expression comparing *Cd68^hi^*/*Ctsd^hi^* MDMs with other MDMs (FDR < 0.05, |log_2_FC| ≥ 0.25). This dataset corresponds to Figure 3D.

Gene	*p*_val	*p*_val_adj	Avg_log_2_FC	pct.1	pct.2
***Ctsb***	2.18462 × 10^−67^	3.83029 × 10^−63^	2.120668212	1	0.991
***Ctsd***	3.40975 × 10^−65^	5.97831 × 10^−61^	2.242470403	1	0.836
***Grn***	9.94594 × 10^−62^	1.74382 × 10^−57^	1.36118394	1	0.96
***Lgmn***	3.99858 × 10^−55^	7.01071 × 10^−51^	1.475010159	1	0.895
***Cd68***	3.7172 × 10^−54^	6.51737 × 10^−50^	1.418655121	1	0.944
***Syngr1***	7.69851 × 10^−53^	1.34978 × 10^−48^	3.139527169	0.876	0.265
***Cd63***	8.61317 × 10^−49^	1.51015 × 10^−44^	2.256296336	0.957	0.481
***Apoe***	1.33234 × 10^−46^	2.33599 × 10^−42^	1.491811175	1	0.864
***Hmox1***	1.62572 × 10^−46^	2.85037 × 10^−42^	2.0218968	0.968	0.676
***Uap1l1***	3.88705 × 10^−46^	6.81517 × 10^−42^	2.067383489	0.93	0.395
***Lhfpl2***	7.25498 × 10^−46^	1.27202 × 10^−41^	2.479636953	0.892	0.281
***Emp1***	1.03096 × 10^−45^	1.80758 × 10^−41^	1.785761344	0.989	0.503
***Psap***	8.37344 × 10^−44^	1.46811 × 10^−39^	1.182018996	1	0.985
***Gpnmb***	1.27898 × 10^−43^	2.24243 × 10^−39^	3.413345094	0.762	0.164
***Fabp5***	5.78356 × 10^−43^	1.01403 × 10^−38^	2.982169672	0.87	0.377
***Vat1***	5.12152 × 10^−42^	8.97956 × 10^−38^	2.383679129	0.87	0.383
***Plin2***	1.03862 × 10^−41^	1.82101 × 10^−37^	1.455717275	1	0.852
***Nrp1***	1.16082 × 10^−41^	2.03527 × 10^−37^	2.086823981	0.914	0.38
***S1pr1***	4.53423 × 10^−41^	7.94986 × 10^−37^	3.00983235	0.665	0.09
***Nrp2***	1.57682 × 10^−40^	2.76465 × 10^−36^	1.720330569	0.957	0.457
***Tpp1***	6.27603 × 10^−40^	1.10038 × 10^−35^	1.331317534	0.962	0.738
***Lamp1***	9.42024 × 10^−40^	1.65165 × 10^−35^	1.048101819	1	0.901
***Lgals3***	4.09726 × 10^−39^	7.18372 × 10^−35^	1.395479005	1	0.917
***Hexa***	1.62423 × 10^−38^	2.84775 × 10^−34^	1.072993135	0.995	0.935
***Cpd***	3.90615 × 10^−38^	6.84866 × 10^−34^	1.530337315	0.951	0.562
***Nav2***	4.26443 × 10^−36^	7.47683 × 10^−32^	2.993524959	0.605	0.086
***Lipa***	8.61251 × 10^−36^	1.51003 × 10^−31^	1.551659365	0.946	0.676
***App***	2.71471 × 10^−35^	4.7597 × 10^−31^	0.882207485	0.995	0.948
***Trem2***	3.34305 × 10^−35^	5.86137 × 10^−31^	1.49126655	0.984	0.515
***Cstb***	4.68393 × 10^−35^	8.21233 × 10^−31^	1.252241197	0.989	0.806
***Ctsz***	1.19872 × 10^−34^	2.10172 × 10^−30^	0.848539594	1	0.975
***Hexb***	1.54731 × 10^−34^	2.7129 × 10^−30^	1.305063117	0.984	0.846
***Pdpn***	2.16727 × 10^−34^	3.79987 × 10^−30^	2.560062559	0.665	0.133
***Nceh1***	3.68821 × 10^−34^	6.46653 × 10^−30^	1.526850084	0.957	0.583
***Timp2***	8.03659 × 10^−34^	1.40905 × 10^−29^	2.565395557	0.789	0.287
***Pf4***	1.79126 × 10^−33^	3.14062 × 10^−29^	2.810500581	0.751	0.216
***C3ar1***	2.28529 × 10^−33^	4.00679 × 10^−29^	1.157102939	0.995	0.704
***Fnip2***	3.34392 × 10^−33^	5.86289 × 10^−29^	1.324767614	0.995	0.682
***Igf2r***	1.02496 × 10^−32^	1.79707 × 10^−28^	1.978614166	0.789	0.259
***Abca1***	1.73662 × 10^−32^	3.04482 × 10^−28^	1.368094226	0.968	0.522
***Rnf128***	8.59725 × 10^−32^	1.50736 × 10^−27^	3.029716697	0.605	0.117
***Osbpl8***	2.03096 × 10^−31^	3.56088 × 10^−27^	1.457811566	0.941	0.614
***Npc1***	2.49797 × 10^−31^	4.37969 × 10^−27^	1.150792799	0.989	0.799
***Plxna1***	2.60408 × 10^−31^	4.56574 × 10^−27^	1.598132953	0.886	0.358
***Bcl2l1***	5.64698 × 10^−31^	9.90085 × 10^−27^	1.549933178	0.919	0.466
***Cd84***	6.65089 × 10^−31^	1.1661× 10^−26^	1.081061753	0.973	0.713
***Slc6a8***	1.65331 × 10^−30^	2.89875 × 10^−26^	2.584196724	0.627	0.148
***Pld3***	2.6382 × 10^−30^	4.62556 × 10^−26^	1.491274478	0.924	0.568
***Stab1***	2.92251 × 10^−30^	5.12404 × 10^−26^	1.481742393	0.973	0.685
***Tspan4***	3.66215 × 10^−30^	6.42084 × 10^−26^	1.978352616	0.795	0.293
***Anxa5***	4.6988 × 10^−30^	8.2384 × 10^−26^	1.127061123	0.951	0.784
***Esyt1***	5.92334 × 10^−30^	1.03854 × 10^−25^	1.078467014	0.968	0.901
***Gla***	1.24977 × 10^−29^	2.19122 × 10^−25^	1.596784694	0.859	0.37
***Mpeg1***	1.30761 × 10^−29^	2.29263 × 10^−25^	0.791895979	1	0.963
***Atp6v1a***	1.33531 × 10^−29^	2.3412 × 10^−25^	1.273026733	0.941	0.654
***Mertk***	1.45137 × 10^−29^	2.54469 × 10^−25^	1.381607958	0.924	0.444
***Lrp1***	1.78373 × 10^−29^	3.12742 × 10^−25^	0.83471313	0.995	0.883
***Mcfd2***	2.50076 × 10^−29^	4.38459 × 10^−25^	1.309917299	0.93	0.682
***Abhd12***	5.2807 × 10^−29^	9.25866 × 10^−25^	1.125776689	0.978	0.818
***Ctsa***	1.32926 × 10^−28^	2.3306 × 10^−24^	0.724466428	1	0.96
***Lonrf3***	2.30548 × 10^−28^	4.04219 × 10^−24^	2.631693156	0.519	0.077
***Sash1***	2.36712 × 10^−28^	4.15027 × 10^−24^	1.416964583	0.903	0.444
***Ms4a7***	2.70286 × 10^−28^	4.73893 × 10^−24^	1.389746612	0.962	0.497
***Papss2***	3.46204 × 10^−28^	6.07 × 10^−24^	1.561606596	0.795	0.272
***Mfsd1***	1.44089 × 10^−27^	2.52631 × 10^−23^	0.955750221	0.973	0.864
***Rnase4***	4.04564 × 10^−27^	7.09322 × 10^−23^	1.285237316	0.951	0.59
***Fblim1***	1.78017 × 10^−26^	3.12117 × 10^−22^	2.23417178	0.573	0.127
***Hpgds***	1.97509 × 10^−26^	3.46292 × 10^−22^	2.003438198	0.659	0.179
***Abcc3***	2.52704 × 10^−26^	4.43065 × 10^−22^	1.155780868	0.968	0.639
***Tmem87b***	3.75203 × 10^−26^	6.57843 × 10^−22^	1.520344614	0.859	0.414
***Atf3***	3.83167 × 10^−26^	6.71807 × 10^−22^	1.69645415	0.708	0.201
***Anxa4***	6.12022 × 10^−26^	1.07306 × 10^−21^	1.440288196	0.87	0.444
***Naglu***	2.5204 × 10^−25^	4.41901 × 10^−21^	1.616263028	0.805	0.364
***Dpp7***	3.71597 × 10^−25^	6.51521 × 10^−21^	1.75757245	0.751	0.284
***Creg1***	4.72167 × 10^−25^	8.2785 × 10^−21^	1.173355784	0.978	0.815
***Ccl7***	5.06179 × 10^−25^	8.87484 × 10^−21^	2.749625489	0.492	0.086
***Tmem106a***	6.63539 × 10^−25^	1.16338 × 10^−20^	1.005848668	0.968	0.775
***Hebp1***	7.28713 × 10^−25^	1.27765 × 10^−20^	2.019805977	0.665	0.204
***Tmem104***	1.236 × 10^−24^	2.16707 × 10^−20^	1.214771689	0.946	0.645
***Gns***	1.44396 × 10^−24^	2.5317 × 10^−20^	0.795859368	0.995	0.904
***Enpp1***	2.36287 × 10^−24^	4.14283 × 10^−20^	1.291165652	0.859	0.352
***Bhlhe41***	3.89341 × 10^−24^	6.82632 × 10^−20^	2.635398416	0.492	0.093
***Slc3a2***	4.54562 × 10^−24^	7.96983 × 10^−20^	0.970708527	0.989	0.778
***Slc27a1***	7.17035 × 10^−24^	1.25718 × 10^−19^	1.852431353	0.735	0.315
***Rhob***	7.18916 × 10^−24^	1.26047 × 10^−19^	1.378985108	0.903	0.491
***Mfsd11***	1.17398 × 10^−23^	2.05833 × 10^−19^	1.404327593	0.854	0.41
***Ctsl***	1.27626 × 10^−23^	2.23767 × 10^−19^	1.151008119	0.995	0.753
***Atp6v1c1***	1.28372 × 10^−23^	2.25075 × 10^−19^	1.196948414	0.903	0.571
***Calr***	1.40501 × 10^−23^	2.4634 × 10^−19^	0.922450032	0.968	0.793
***Arg1***	1.41538 × 10^−23^	2.48159 × 10^−19^	1.465630609	0.8	0.358
***Capg***	1.47916 × 10^−23^	2.5934 × 10^−19^	1.046083917	0.962	0.627
***Tnfsf12***	1.81226 × 10^−23^	3.17743 × 10^−19^	1.316747337	0.876	0.519
***Sdcbp***	3.46976 × 10^−23^	6.08353 × 10^−19^	0.709866322	0.995	0.96
***Gpr157***	3.99443 × 10^−23^	7.00343 × 10^−19^	2.244184284	0.53	0.117
***Tex264***	4.59828 × 10^−23^	8.06217 × 10^−19^	1.584862069	0.735	0.29
***Rasgef1b***	5.04045 × 10^−23^	8.83742 × 10^−19^	1.436000214	0.719	0.256
***Itga6***	6.84418 × 10^−23^	1.19999 × 10^−18^	1.232089408	0.897	0.503
***Por***	9.02385 × 10^−23^	1.58215 × 10^−18^	1.147226807	0.946	0.636
***Sypl***	1.18411 × 10^−22^	2.0761 × 10^−18^	0.938480827	0.957	0.722
***Gnptab***	1.38601 × 10^−22^	2.4301 × 10^−18^	1.332424268	0.865	0.543
***Ly9***	1.39362 × 10^−22^	2.44343 × 10^−18^	1.567365747	0.697	0.259
***Cd180***	1.7302 × 10^−22^	3.03356 × 10^−18^	1.396627961	0.822	0.386
***Slc48a1***	2.78462 × 10^−22^	4.88228 × 10^−18^	1.652552871	0.773	0.423
***Tmem37***	3.12057 × 10^−22^	5.47129 × 10^−18^	1.052758833	0.919	0.472
***Lgals1***	3.22033 × 10^−22^	5.6462 × 10^−18^	1.443757675	0.762	0.33
***Adgre1***	4.34106 × 10^−22^	7.61119 × 10^−18^	0.990770802	0.968	0.645
***Spp1***	5.03777 × 10^−22^	8.83271 × 10^−18^	2.212041132	0.811	0.512
***Bcar3***	5.69172 × 10^−22^	9.9793 × 10^−18^	3.068954812	0.384	0.049
***Glmp***	6.64128 × 10^−22^	1.16441 × 10^−17^	0.828931958	0.989	0.83
***Tent5c***	6.65979 × 10^−22^	1.16766 × 10^−17^	1.645794578	0.632	0.185
***Gaa***	1.00992 × 10^−21^	1.77069 × 10^−17^	1.201169935	0.881	0.546
***Slc7a8***	1.05084 × 10^−21^	1.84243 × 10^−17^	1.018231855	0.957	0.586
***Mpp1***	1.63149 × 10^−21^	2.8605 × 10^−17^	1.114302561	0.924	0.546
***Cebpa***	2.31856 × 10^−21^	4.06514 × 10^−17^	1.106197908	0.93	0.676
***Pdia6***	4.3261 × 10^−21^	7.58495 × 10^−17^	0.860133868	0.973	0.855
***Apoc2***	1.08712 × 10^−20^	1.90605 × 10^−16^	0.954726838	0.968	0.608
***Ang***	1.53991 × 10^−20^	2.69992 × 10^−16^	1.427514375	0.735	0.269
***Gna12***	1.70503 × 10^−20^	2.98943 × 10^−16^	0.937539283	0.946	0.633
***Htr2b***	2.16608 × 10^−20^	3.7978 × 10^−16^	4.456220306	0.286	0.015
***Rab7***	2.52376 × 10^−20^	4.42491 × 10^−16^	0.804947475	0.968	0.83
***Atp6v0c***	2.79169 × 10^−20^	4.89466 × 10^−16^	0.58547998	0.989	0.944
***Gga2***	2.84017 × 10^−20^	4.97966 × 10^−16^	1.738982073	0.616	0.194
***Gde1***	3.25682 × 10^−20^	5.71019 × 10^−16^	0.918568775	0.962	0.648
***Arhgap10***	4.52819 × 10^−20^	7.93927 × 10^−16^	1.740229225	0.654	0.231
***Osbpl11***	5.57853 × 10^−20^	9.78084 × 10^−16^	1.358303149	0.822	0.417
***Tceal9***	8.93016 × 10^−20^	1.56572 × 10^−15^	1.62999134	0.6	0.179
***Pon3***	1.30244 × 10^−19^	2.28357 × 10^−15^	2.105910455	0.524	0.151
***Tmem86a***	1.30678 × 10^−19^	2.29118 × 10^−15^	0.889948363	0.962	0.667
***Pdia4***	1.84988 × 10^−19^	3.24339 × 10^−15^	1.009534388	0.924	0.694
***Cipc***	2.3605 × 10^−19^	4.13867 × 10^−15^	1.588389987	0.568	0.17
***Canx***	2.69357 × 10^−19^	4.72263 × 10^−15^	0.792472991	0.973	0.79
***Comtd1***	3.13989 × 10^−19^	5.50517 × 10^−15^	1.883500627	0.551	0.154
***Sgk1***	6.41776 × 10^−19^	1.12523 × 10^−14^	1.101081959	0.941	0.617
***Gas2l3***	7.52083 × 10^−19^	1.31863 × 10^−14^	1.481417566	0.643	0.213
***Nek6***	1.30517 × 10^−18^	2.28836 × 10^−14^	1.086251993	0.876	0.457
***Sgpl1***	1.45642 × 10^−18^	2.55354 × 10^−14^	0.643144935	0.984	0.91
***Myof***	2.84059 × 10^−18^	4.9804 × 10^−14^	1.159798946	0.832	0.435
***Flvcr1***	2.92204 × 10^−18^	5.12322 × 10^−14^	1.247084756	0.8	0.395
***Dtnbp1***	2.92372 × 10^−18^	5.12617 × 10^−14^	1.05316515	0.876	0.537
***Clcn7***	4.78989 × 10^−18^	8.39812 × 10^−14^	1.054863352	0.903	0.568
***Ctsh***	5.26375 × 10^−18^	9.22894 × 10^−14^	0.590568039	0.989	0.975
***Ccl2***	5.59203 × 10^−18^	9.8045 × 10^−14^	2.063735227	0.632	0.262
***Appl2***	1.50041 × 10^−17^	2.63068 × 10^−13^	1.478085142	0.659	0.259
***Wls***	2.42459 × 10^−17^	4.25104 × 10^−13^	1.030809822	0.843	0.417
***Renbp***	3.08046 × 10^−17^	5.40096 × 10^−13^	1.164988286	0.768	0.398
***Akr1a1***	3.14134 × 10^−17^	5.50772 × 10^−13^	0.717688507	0.989	0.954
***Vps26a***	3.62032 × 10^−17^	6.3475 × 10^−13^	0.831507683	0.941	0.75
***Jag1***	4.8504 × 10^−17^	8.5042 × 10^−13^	2.77475526	0.405	0.096
***Pqlc3***	6.84975 × 10^−17^	1.20097 × 10^−12^	1.019864257	0.849	0.429
***Plec***	7.60464 × 10^−17^	1.33332 × 10^−12^	0.768651595	0.968	0.735
***Tnfsf13***	8.78662 × 10^−17^	1.54056 × 10^−12^	0.987946414	0.892	0.58
***Prdx1***	1.11198 × 10^−16^	1.94963 × 10^−12^	1.018348765	0.914	0.642
***Cd36***	1.14742 × 10^−16^	2.01177 × 10^−12^	2.674167093	0.4	0.09
***Abcg1***	1.19749 × 10^−16^	2.09955 × 10^−12^	0.990001054	0.87	0.435
***Cndp2***	1.53266 × 10^−16^	2.68721 × 10^−12^	0.862204942	0.941	0.627
***Qk***	1.57272 × 10^−16^	2.75745 × 10^−12^	0.683078427	0.968	0.914
***Fmn1***	1.63844 × 10^−16^	2.87267 × 10^−12^	1.56614922	0.584	0.213
***Itsn1***	1.70593 × 10^−16^	2.991 × 10^−12^	1.126936872	0.686	0.284
***Bcl2a1b***	1.71469 × 10^−16^	3.00636 × 10^−12^	1.423351993	0.611	0.238
***Rxra***	1.91599 × 10^−16^	3.35931 × 10^−12^	1.17398708	0.773	0.392
***Blnk***	2.07546 × 10^−16^	3.6389 × 10^−12^	2.194009294	0.395	0.09
***Hspa5***	2.15806 × 10^−16^	3.78373 × 10^−12^	0.854788832	0.946	0.799
***Hs1bp3***	2.26337 × 10^−16^	3.96837 × 10^−12^	1.530476735	0.568	0.198
***Zfp704***	2.47341 × 10^−16^	4.33663 × 10^−12^	2.3430807	0.405	0.099
***Ap1b1***	2.96959 × 10^−16^	5.20659 × 10^−12^	0.909108947	0.876	0.54
***Cyba***	8.23141 × 10^−16^	1.44321 × 10^−11^	0.46715885	0.995	0.978
***Adam8***	8.74615 × 10^−16^	1.53346 × 10^−11^	0.809583834	0.941	0.599
***Slc43a2***	9.47392 × 10^−16^	1.66106 × 10^−11^	0.647273824	0.984	0.821
***Gba***	9.75549 × 10^−16^	1.71043 × 10^−11^	0.845930919	0.93	0.691
***Cd93***	9.92825 × 10^−16^	1.74072 × 10^−11^	0.72958028	0.978	0.75
***Cd9***	1.03181 × 10^−15^	1.80907 × 10^−11^	1.236157994	0.735	0.389
***Tns1***	1.30105 × 10^−15^	2.28114 × 10^−11^	2.372648601	0.335	0.062
***Mtss1***	1.70341 × 10^−15^	2.98659 × 10^−11^	1.537763616	0.551	0.198
***Ell2***	1.98615 × 10^−15^	3.48232 × 10^−11^	1.446885757	0.686	0.336
***Man1c1***	2.23034 × 10^−15^	3.91045 × 10^−11^	1.239243068	0.741	0.386
***Ckap4***	2.30109 × 10^−15^	4.03451 × 10^−11^	1.128725066	0.843	0.552
***Atp6ap2***	2.44083 × 10^−15^	4.2795 × 10^−11^	0.718137053	0.951	0.833
***Gclm***	2.63076 × 10^−15^	4.61251 × 10^−11^	1.775358105	0.686	0.349
***Bst2***	2.93966 × 10^−15^	5.1541 × 10^−11^	0.998659763	0.876	0.611
***Fcrls***	3.40934 × 10^−15^	5.97759 × 10^−11^	0.832080514	0.632	0.253
***Osbpl1a***	3.48312 × 10^−15^	6.10695 × 10^−11^	1.257537552	0.551	0.176
***Slc38a6***	3.50303 × 10^−15^	6.14186 × 10^−11^	1.544937472	0.589	0.213
***Vps35***	4.16501 × 10^−15^	7.30251× 10^−11^	0.828588021	0.924	0.688
***Lmna***	4.18923 × 10^−15^	7.34498 × 10^−11^	1.038031853	0.686	0.293
***Sh3bp5***	4.46232 × 10^−15^	7.82379 × 10^−11^	1.179387006	0.638	0.256
***Ctss***	4.48783 × 10^−15^	7.86852 × 10^−11^	0.442487048	1	0.994
***Sec14l1***	4.64793 × 10^−15^	8.14922 × 10^−11^	1.050517812	0.832	0.528
***Cbr2***	9.13786 × 10^−15^	1.60214 × 10^−10^	2.822541259	0.335	0.071
***Tmem140***	9.90704 × 10^−15^	1.737 × 10^−10^	1.514705899	0.47	0.145
***Lat2***	1.163 × 10^−14^	2.03908 × 10^−10^	0.765109718	0.968	0.762
***Atp2a2***	1.47449 × 10^−14^	2.58522 × 10^−10^	0.656132206	0.968	0.79
***Tnfaip2***	1.50152 × 10^−14^	2.63261 × 10^−10^	1.184448383	0.703	0.327
***Sirpa***	1.84449 × 10^−14^	3.23395 × 10^−10^	0.51355163	0.995	0.932
***Rftn1***	1.96487 × 10^−14^	3.44501 × 10^−10^	0.964736137	0.849	0.491
***Hgf***	2.36432 × 10^−14^	4.14537 × 10^−10^	1.053608691	0.692	0.312
***Hk3***	2.45427 × 10^−14^	4.30307 × 10^−10^	1.336703749	0.703	0.33
***Cxcl16***	3.02991 × 10^−14^	5.31234 × 10^−10^	0.539816586	0.935	0.586
***Gas6***	3.19299 × 10^−14^	5.59828 × 10^−10^	2.591078376	0.362	0.096
***Ppib***	3.87232 × 10^−14^	6.78933 × 10^−10^	0.553141372	1	0.926
***Cxcr4***	4.11432 × 10^−14^	7.21364 × 10^−10^	0.628462869	0.957	0.704
***Steap3***	4.15009 × 10^−14^	7.27635 × 10^−10^	1.760971736	0.357	0.08
***Tatdn2***	4.20965 × 10^−14^	7.38078 × 10^−10^	0.697509265	0.941	0.735
***Mgat5***	4.94689 × 10^−14^	8.67339 × 10^−10^	0.831803509	0.897	0.608
***S1pr2***	5.0749 × 10^−14^	8.89782 × 10^−10^	1.024946516	0.768	0.367
***Man2b2***	5.37075 × 10^−14^	9.41654 × 10^−10^	0.933668389	0.838	0.611
***Il10rb***	5.69431 × 10^−14^	9.98384 × 10^−10^	0.621839883	0.984	0.873
***Rab1a***	6.02408 × 10^−14^	1.0562 × 10^−9^	0.618304876	0.957	0.892
***Gdpd1***	6.02481 × 10^−14^	1.05633 × 10^−9^	2.556964134	0.276	0.043
***Rgl1***	6.18523 × 10^−14^	1.08446 × 10^−9^	0.941596079	0.773	0.398
***Gsn***	6.88982 × 10^−14^	1.20799 × 10^−9^	0.758777817	0.951	0.775
***Naa50***	7.88605 × 10^−14^	1.38266 × 10^−9^	0.925736858	0.854	0.528
***Crtap***	8.04968 × 10^−14^	1.41135 × 10^−9^	1.270657096	0.622	0.272
***Slc35f6***	8.22235 × 10^−14^	1.44162 × 10^−9^	0.722244047	0.962	0.698
***Pgpep1***	8.42374 × 10^−14^	1.47693 × 10^−9^	1.548465185	0.389	0.096
***Galns***	8.83641 × 10^−14^	1.54929 × 10^−9^	0.946266343	0.805	0.448
***Kif3a***	9.22072 × 10^−14^	1.61667 × 10^−9^	2.186563669	0.351	0.083
***Lrp12***	9.70184 × 10^−14^	1.70102 × 10^−9^	1.619385044	0.53	0.204
***Grina***	1.31307 × 10^−13^	2.30221 × 10^−9^	0.693637342	0.968	0.79
***Pdia3***	1.3883 × 10^−13^	2.4341 × 10^−9^	0.609888113	0.973	0.892
***Ddx1***	1.54141 × 10^−13^	2.70255 × 10^−9^	1.081076708	0.611	0.247
***Hvcn1***	2.11072 × 10^−13^	3.70072 × 10^−9^	1.314245814	0.503	0.173
***Mroh1***	2.16985 × 10^−13^	3.8044 × 10^−9^	0.84998661	0.859	0.506
***Ecm1***	2.33301 × 10^−13^	4.09046 × 10^−9^	0.639565274	0.995	0.744
***Hsp90b1***	2.33562 × 10^−13^	4.09504 × 10^−9^	0.660094583	0.962	0.83
***Ninj1***	2.39945 × 10^−13^	4.20695 × 10^−9^	0.859149771	0.897	0.574
***Mdfic***	2.76151 × 10^−13^	4.84175 × 10^−9^	0.696434005	0.957	0.722
***Tuba1c***	3.12708 × 10^−13^	5.48272 × 10^−9^	0.814924388	0.903	0.633
***Mylip***	3.69632 × 10^−13^	6.48075 × 10^−9^	0.984079431	0.719	0.346
***Ywhag***	3.95153 × 10^−13^	6.92822 × 10^−9^	0.691318998	0.951	0.796
***C1qc***	4.04884 × 10^−13^	7.09884 × 10^−9^	1.17719065	0.697	0.42
***Aga***	4.14351 × 10^−13^	7.26482 × 10^−9^	1.472381848	0.497	0.176
***Fth1***	4.28541 × 10^−13^	7.51361 × 10^−9^	0.691494245	1	0.981
***Dennd4c***	4.64882 × 10^−13^	8.15078 × 10^−9^	0.843487743	0.897	0.623
***Angptl4***	4.70945 × 10^−13^	8.25708 × 10^−9^	2.457847254	0.314	0.068
***Snx27***	4.78145 × 10^−13^	8.38332 × 10^−9^	1.097842307	0.741	0.42
***Plod1***	5.44606 × 10^−13^	9.54858 × 10^−9^	1.020550419	0.784	0.454
***Cyb5r1***	7.15328 × 10^−13^	1.25419 × 10^−8^	0.895153867	0.811	0.42
***Paqr7***	8.60085 × 10^−13^	1.50799 × 10^−8^	1.118024243	0.654	0.309
***Blvrb***	1.08979 × 10^−12^	1.91072 × 10^−8^	1.407671376	0.595	0.275
***Tmem8***	1.17702 × 10^−12^	2.06367 × 10^−8^	1.416257121	0.546	0.228
***Dmxl2***	1.19455 × 10^−12^	2.0944 × 10^−8^	0.770733652	0.843	0.5
***Itm2b***	1.29724 × 10^−12^	2.27445 × 10^−8^	0.423105897	1	0.985
***Bax***	1.35625 × 10^−12^	2.37791 × 10^−8^	0.68496962	0.935	0.747
***Cln8***	1.55714 × 10^−12^	2.73013 × 10^−8^	0.749872128	0.924	0.623
***Hyal1***	1.87679 × 10^−12^	3.29057 × 10^−8^	3.126581298	0.222	0.028
***Frmd4b***	1.99491 × 10^−12^	3.49768 × 10^−8^	0.949566468	0.643	0.284
***Iqgap1***	2.15228 × 10^−12^	3.77359 × 10^−8^	0.546073826	0.978	0.96
***Pfkfb3***	2.19803 × 10^−12^	3.85381 × 10^−8^	0.991066065	0.811	0.488
***Ahnak2***	2.24307 × 10^−12^	3.93278 × 10^−8^	1.363912504	0.314	0.071
***Eea1***	2.79874 × 10^−12^	4.90702 × 10^−8^	1.003108018	0.719	0.389
***Idua***	2.82959 × 10^−12^	4.96112 × 10^−8^	1.041191885	0.692	0.361
***Bri3***	2.87058 × 10^−12^	5.03299 × 10^−8^	0.680758298	0.941	0.731
***Camk1***	3.19147 × 10^−12^	5.59561 × 10^−8^	0.950847306	0.811	0.469
***Atp6v0a1***	3.38687 × 10^−12^	5.9382 × 10^−8^	0.794105003	0.859	0.62
***Dot1l***	3.79523 × 10^−12^	6.65418 × 10^−8^	1.220001783	0.557	0.228
***Soat1***	4.15701 × 10^−12^	7.28848 × 10^−8^	0.574030757	0.968	0.833
***F7***	4.20789 × 10^−12^	7.37769 × 10^−8^	2.065604851	0.368	0.114
***Adgrl2***	4.60565 × 10^−12^	8.07508 × 10^−8^	1.050616021	0.4	0.117
***Nampt***	4.76752 × 10^−12^	8.35889 × 10^−8^	0.818971062	0.816	0.5
***Snx8***	4.8822 × 10^−12^	8.55996 × 10^−8^	1.013362224	0.768	0.429
***Hsd3b7***	5.22455 × 10^−12^	9.16021 × 10^−8^	1.154856309	0.665	0.336
***Dmxl1***	5.25063 × 10^−12^	9.20593 × 10^−8^	1.028686574	0.714	0.373
***C1qb***	5.7844 × 10^−12^	1.01418 × 10^−7^	1.06534189	0.741	0.522
***Coprs***	5.82122 × 10^−12^	1.02063 × 10^−7^	2.199862442	0.292	0.065
***Folr2***	6.23441 × 10^−12^	1.09308 × 10^−7^	2.767121203	0.265	0.052
***Spg20***	6.59476 × 10^−12^	1.15626 × 10^−7^	1.602095086	0.357	0.096
***B4galt1***	6.64174 × 10^−12^	1.1645 × 10^−7^	0.558327557	0.984	0.836
***Atp6ap1***	7.67567 × 10^−12^	1.34577 × 10^−7^	0.49269663	0.995	0.944
***Stx4a***	8.16958 × 10^−12^	1.43237 × 10^−7^	0.74878208	0.892	0.688
***Dusp3***	8.33685 × 10^−12^	1.4617 × 10^−7^	0.640754102	0.962	0.833
***Txnrd1***	9.0023 × 10^−12^	1.57837 × 10^−7^	0.746731772	0.822	0.491
***Il7r***	9.29679 × 10^−12^	1.63001 × 10^−7^	0.910698891	0.443	0.151
***Zfand2a***	9.39852 × 10^−12^	1.64784 × 10^−7^	1.143956948	0.492	0.185
***Cd24a***	9.70199 × 10^−12^	1.70105 × 10^−7^	0.853691508	0.659	0.315
***Slc7a7***	1.08561 × 10^−11^	1.90339 × 10^−7^	0.902076227	0.822	0.515
***Zranb3***	1.1043 × 10^−11^	1.93617 × 10^−7^	3.203133226	0.216	0.031
***Tmem65***	1.1115 × 10^−11^	1.94879 × 10^−7^	0.947232982	0.784	0.485
***Slc37a2***	1.11476 × 10^−11^	1.95451 × 10^−7^	0.819883393	0.865	0.54
***Gusb***	1.52005 × 10^−11^	2.6651 × 10^−7^	0.497363803	0.978	0.898
***Atp6v1g1***	2.11818 × 10^−11^	3.71381 × 10^−7^	0.696651836	0.93	0.778
***Actn1***	2.53558 × 10^−11^	4.44562 × 10^−7^	0.739415653	0.508	0.191
***Plau***	2.82854 × 10^−11^	4.95927 × 10^−7^	1.074508104	0.659	0.336
***Cyth1***	2.85809 × 10^−11^	5.0111 × 10^−7^	0.775183729	0.892	0.67
***Jade2***	2.94785 × 10^−11^	5.16847 × 10^−7^	2.292294284	0.227	0.037
***Cd48***	2.98375 × 10^−11^	5.23141 × 10^−7^	0.622685817	0.941	0.849
***Npc2***	3.0108 × 10^−11^	5.27884 × 10^−7^	0.384049993	0.995	0.966
***Slc11a1***	3.13018 × 10^−11^	5.48815 × 10^−7^	0.641887221	0.962	0.731
***Prokr1***	3.2203 × 10^−11^	5.64615 × 10^−7^	2.776865172	0.2	0.025
***Prmt2***	3.46804 × 10^−11^	6.08052 × 10^−7^	1.796500167	0.324	0.086
***Amdhd2***	3.67989 × 10^−11^	6.45195 × 10^−7^	0.878480171	0.773	0.457
***P2ry2***	3.71165 × 10^−11^	6.50764 × 10^−7^	0.917185351	0.416	0.13
***Tns3***	3.96984 × 10^−11^	6.96032 × 10^−7^	0.591398865	0.908	0.596
***Fuca2***	5.26239 × 10^−11^	9.22654 × 10^−7^	0.67246889	0.935	0.762
***Plxnc1***	5.27128 × 10^−11^	9.24214 × 10^−7^	0.839332209	0.714	0.386
***Tubb4b***	5.29151 × 10^−11^	9.2776 × 10^−7^	0.886311513	0.724	0.426
***Coro1c***	5.7608 × 10^−11^	1.01004 × 10^−6^	0.583665382	0.897	0.654
***Gal3st4***	5.79697 × 10^−11^	1.01638 × 10^−6^	2.82450823	0.189	0.022
***Rin2***	6.25717 × 10^−11^	1.09707 × 10^−6^	0.671094047	0.865	0.497
***Rap2a***	6.69548 × 10^−11^	1.17392 × 10^−6^	0.899670487	0.686	0.386
***Dgkz***	7.49156 × 10^−11^	1.3135 × 10^−6^	0.571272046	0.962	0.843
***Cpeb2***	8.61797 × 10^−11^	1.51099 × 10^−6^	0.938779149	0.762	0.448
***Naa20***	8.9919 × 10^−11^	1.57655 × 10^−6^	0.800945722	0.811	0.472
***Rai14***	9.82077 × 10^−11^	1.72188 × 10^−6^	0.648683909	0.914	0.559
***Fam20c***	9.918 × 10^−11^	1.73892 × 10^−6^	1.006564069	0.811	0.494
***C1qa***	1.00469 × 10^−10^	1.76153 × 10^−6^	0.993315968	0.665	0.398
***Kcnk13***	1.09459 × 10^−10^	1.91914 × 10^−6^	0.932312197	0.589	0.259
***Sdf2l1***	1.09808 × 10^−10^	1.92526 × 10^−6^	0.690223382	0.897	0.701
***Serinc1***	1.10555 × 10^−10^	1.93836 × 10^−6^	0.505101959	0.962	0.861
***Slc23a2***	1.15138 × 10^−10^	2.01872 × 10^−6^	0.893185269	0.768	0.441
***Txn1***	1.15808 × 10^−10^	2.03046 × 10^−6^	0.853463815	0.822	0.519
***Rap2b***	1.21639 × 10^−10^	2.13269 × 10^−6^	0.679382174	0.935	0.688
***Slc38a7***	1.41927 × 10^−10^	2.4884 × 10^−6^	0.901778938	0.627	0.309
***Ldlrap1***	1.4614 × 10^−10^	2.56228 × 10^−6^	0.751231774	0.838	0.509
***Lrpap1***	1.47184 × 10^−10^	2.58058 × 10^−6^	0.5479928	0.946	0.728
***Rnasek***	1.47824 × 10^−10^	2.5918 × 10^−6^	0.670077248	0.908	0.793
***Fabp3***	1.48928 × 10^−10^	2.61115 × 10^−6^	2.415172846	0.168	0.015
***St6galnac4***	1.5281 × 10^−10^	2.67921 × 10^−6^	0.860589131	0.8	0.481
***Tlr13***	1.76601 × 10^−10^	3.09634 × 10^−6^	0.500115452	0.973	0.858
***Rragc***	1.82458 × 10^−10^	3.19903 × 10^−6^	0.747300332	0.886	0.636
***Plekhm1***	2.13501 × 10^−10^	3.74331 × 10^−6^	1.183461813	0.632	0.33
***Tpcn2***	2.60707 × 10^−10^	4.57097 × 10^−6^	0.770971587	0.768	0.417
***Arhgap19***	2.62082 × 10^−10^	4.59508 × 10^−6^	2.068553284	0.243	0.049
***Dnmt1***	2.69565 × 10^−10^	4.72629 × 10^−6^	1.003936655	0.659	0.377
***Mitf***	2.90446 × 10^−10^	5.09238 × 10^−6^	0.904438696	0.584	0.256
***Nlrc3***	3.16618 × 10^−10^	5.55126 × 10^−6^	3.482605193	0.189	0.028
***Pltp***	3.19552 × 10^−10^	5.60271 × 10^−6^	1.068291011	0.757	0.478
***Dhrs3***	3.56628 × 10^−10^	6.25276 × 10^−6^	0.503592336	0.978	0.867
***Cd200r4***	3.84476 × 10^−10^	6.74103 × 10^−6^	1.711798576	0.297	0.077
***Dennd1a***	3.95927 × 10^−10^	6.9418 × 10^−6^	0.689560578	0.735	0.429
***Vps29***	3.96515 × 10^−10^	6.9521 × 10^−6^	0.757293683	0.778	0.46
***Mcoln1***	4.15724 × 10^−10^	7.28888 × 10^−6^	0.935923176	0.735	0.414
***Washc5***	4.16166 × 10^−10^	7.29664 × 10^−6^	0.981563659	0.724	0.429
***Arl8a***	4.59451 × 10^−10^	8.05555 × 10^−6^	0.576215977	0.914	0.719
***Trim47***	4.6601 × 10^−10^	8.17055 × 10^−6^	1.248873868	0.33	0.096
***Rnh1***	4.86513 × 10^−10^	8.53003 × 10^−6^	0.532944904	0.968	0.886
***Lamc1***	5.46695 × 10^−10^	9.58521 × 10^−6^	1.117388542	0.438	0.167
***Ctsk***	5.56485 × 10^−10^	9.75684 × 10^−6^	1.878733322	0.238	0.049
***Myo5a***	6.03968 × 10^−10^	1.05894 × 10^−5^	0.541894146	0.957	0.765
***Plek***	6.06444 × 10^−10^	1.06328 × 10^−5^	0.544354369	0.941	0.852
***Scarb2***	6.24291 × 10^−10^	1.09457 × 10^−5^	0.544791901	0.957	0.858
***Tubb5***	6.55012 × 10^−10^	1.14843 × 10^−5^	0.791018807	0.843	0.617
***Thbs1***	6.91671 × 10^−10^	1.21271 × 10^−5^	0.643205741	0.995	0.71
***Fam129b***	7.03197 × 10^−10^	1.23292 × 10^−5^	0.431964277	0.968	0.71
***Cpeb4***	8.01048 × 10^−10^	1.40448 × 10^−5^	1.115979619	0.676	0.383
***Nr1h3***	9.42609 × 10^−10^	1.65268 × 10^−5^	1.207504167	0.432	0.167
***Psmd1***	9.91828 × 10^−10^	1.73897 × 10^−5^	0.715194471	0.849	0.62
***Mrtfb***	1.01052 × 10^−9^	1.77174 × 10^−5^	1.018537509	0.6	0.306
***Gclc***	1.02458 × 10^−9^	1.79639 × 10^−5^	1.13543266	0.486	0.213
***Galc***	1.03885 × 10^−9^	1.82142 × 10^−5^	0.717434483	0.708	0.367
***Spsb4***	1.08082 × 10^−9^	1.89501 × 10^−5^	2.699348614	0.173	0.022
***Rbm47***	1.11093 × 10^−9^	1.94779 × 10^−5^	0.635993506	0.93	0.685
***Rab3il1***	1.11564 × 10^−9^	1.95605 × 10^−5^	0.539983766	0.962	0.75
***Lamp2***	1.15112 × 10^−9^	2.01826 × 10^−5^	0.351148779	0.989	0.957
***Basp1***	1.1736 × 10^−9^	2.05768 × 10^−5^	0.726122467	0.741	0.451
***Slc41a2***	1.2164 × 10^−9^	2.13271 × 10^−5^	0.955523251	0.465	0.191
***Abcc1***	1.26208 × 10^−9^	2.2128 × 10^−5^	0.807737475	0.638	0.318
***Wdr81***	1.26504 × 10^−9^	2.218 × 10^−5^	0.772260934	0.849	0.559
***Clta***	1.28152 × 10^−9^	2.24689 × 10^−5^	0.414366424	0.995	0.951
***Zdhhc14***	1.3205 × 10^−9^	2.31524 × 10^−5^	1.579162193	0.303	0.086
***Serpinb6a***	1.4396 × 10^−9^	2.52406 × 10^−5^	2.39740478	0.281	0.08
***Pgam1***	1.47949 × 10^−9^	2.59398 × 10^−5^	0.745939107	0.827	0.59
***Ophn1***	1.54236 × 10^−9^	2.70422 × 10^−5^	0.874503239	0.578	0.253
***Vegfb***	1.57411 × 10^−9^	2.75989 × 10^−5^	1.381500576	0.373	0.127
***Sco2***	1.62467 × 10^−9^	2.84854 × 10^−5^	1.111460185	0.514	0.228
***Wdfy3***	1.62997 × 10^−9^	2.85782 × 10^−5^	0.555009779	0.924	0.747
***Hip1***	1.66626 × 10^−9^	2.92145 × 10^−5^	0.510932842	0.93	0.673
***Lonrf1***	1.67869 × 10^−9^	2.94325 × 10^−5^	1.316228317	0.389	0.139
***Yif1b***	1.7616 × 10^−9^	3.08861 × 10^−5^	0.916414724	0.638	0.34
***Gdf15***	1.98015 × 10^−9^	3.4718 × 10^−5^	2.899762006	0.195	0.034
***Acads***	2.01791 × 10^−9^	3.53801 × 10^−5^	1.134729705	0.508	0.235
***Homer3***	2.06895 × 10^−9^	3.62749 × 10^−5^	0.863620794	0.578	0.272
***Tnfrsf26***	2.08483 × 10^−9^	3.65533 × 10^−5^	2.786842635	0.168	0.022
***Arg2***	2.27173 × 10^−9^	3.98302 × 10^−5^	0.877576829	0.384	0.133
***Clcn5***	2.34113 × 10^−9^	4.1047 × 10^−5^	0.847781336	0.627	0.327
***Atp6v1h***	2.6955 × 10^−9^	4.72602 × 10^−5^	0.729868678	0.838	0.559
***Eif1a***	2.71968 × 10^−9^	4.76842 × 10^−5^	0.830360344	0.697	0.429
***M6pr***	2.85692 × 10^−9^	5.00904 × 10^−5^	0.466259814	0.962	0.886
***Lmbrd2***	3.00371 × 10^−9^	5.26641 × 10^−5^	0.980720184	0.557	0.278
***Slc17a5***	3.17155 × 10^−9^	5.56068 × 10^−5^	1.03123946	0.519	0.228
***Nucb1***	3.80309 × 10^−9^	6.66796 × 10^−5^	0.562662445	0.93	0.778
***Cln5***	3.8039 × 10^−9^	6.66938 × 10^−5^	0.726299214	0.816	0.512
***Arhgap24***	3.90748 × 10^−9^	6.85098 × 10^−5^	1.005644925	0.546	0.265
***Aldh2***	4.07904 × 10^−9^	7.15178 × 10^−5^	0.729803781	0.87	0.741
***Selenof***	4.11913 × 10^−9^	7.22207 × 10^−5^	0.645639147	0.854	0.608
***Stra6l***	4.41188 × 10^−9^	7.73534 × 10^−5^	1.434000882	0.238	0.056
***Nr1d2***	4.6452 × 10^−9^	8.14444 × 10^−5^	1.177176865	0.297	0.09
***Ncapg2***	4.7297 × 10^−9^	8.29258 × 10^−5^	0.834319249	0.6	0.296
***Xylt2***	6.12213 × 10^−9^	0.000107339	0.806409665	0.622	0.293
***Rusc2***	6.38521 × 10^−9^	0.000111952	1.212493072	0.411	0.16
***March8***	6.39534 × 10^−9^	0.000112129	1.135519066	0.497	0.225
***Hspd1***	7.10273 × 10^−9^	0.000124532	0.800086569	0.616	0.296
***Slc6a6***	7.24425 × 10^−9^	0.000127013	0.361487959	0.984	0.914
***Vcp***	7.24477 × 10^−9^	0.000127023	0.470461107	0.924	0.781
***Slamf7***	8.42113 × 10^−9^	0.000147648	0.462314549	0.573	0.262
***Arfgef2***	8.56372 × 10^−9^	0.000150148	0.987569276	0.6	0.299
***Cct3***	8.72373 × 10^−9^	0.000152953	0.902581013	0.708	0.423
***Fbxo22***	9.11906 × 10^−9^	0.000159884	0.960753352	0.551	0.265
***Plin3***	9.25158 × 10^−9^	0.000162208	0.999977429	0.589	0.309
***Aldoa***	9.6079 × 10^−9^	0.000168455	0.495574131	0.935	0.833
***Nagk***	1.00905 × 10^−8^	0.000176917	0.897821134	0.541	0.259
***Sirt2***	1.06092 × 10^−8^	0.000186012	0.634951847	0.805	0.549
***Plbd2***	1.08528 × 10^−8^	0.000190282	0.63124313	0.892	0.694
***Selenos***	1.1117 × 10^−8^	0.000194915	0.597087583	0.832	0.571
***Specc1***	1.12548 × 10^−8^	0.000197331	0.656052373	0.697	0.373
***Snx12***	1.20811 × 10^−8^	0.000211817	0.861262688	0.719	0.457
***Sept8***	1.23653 × 10^−8^	0.000216801	0.827072705	0.649	0.358
***Ipo5***	1.26921 × 10^−8^	0.000222531	0.828701417	0.735	0.485
***Vps8***	1.28751 × 10^−8^	0.000225739	0.749345162	0.654	0.349
***Lgals9***	1.3535 × 10^−8^	0.000237309	0.527104013	0.908	0.667
***Sephs2***	1.37255 × 10^−8^	0.000240649	0.701407632	0.854	0.617
***Selenop***	1.41451 × 10^−8^	0.000248005	0.3130667	0.973	0.809
***Litaf***	1.4839 × 10^−8^	0.000260173	0.518280098	0.924	0.759
***Cyp20a1***	1.55928 × 10^−8^	0.000273389	0.941655866	0.584	0.281
***Tfpi***	1.61015 × 10^−8^	0.000282308	1.855716522	0.184	0.034
***Hyal2***	1.62424 × 10^−8^	0.000284778	1.409509337	0.303	0.096
***Atp6v0d1***	1.64568 × 10^−8^	0.000288537	0.491142667	0.914	0.796
***Tmem120a***	1.66039 × 10^−8^	0.000291117	0.992560831	0.497	0.231
***Mmp14***	1.67056 × 10^−8^	0.0002929	0.471762514	0.924	0.691
***Iqsec1***	1.69271 × 10^−8^	0.000296783	0.656404356	0.822	0.552
***Dnajb11***	1.69902 × 10^−8^	0.000297888	0.537665474	0.914	0.812
***Rab20***	1.71724 × 10^−8^	0.000301084	0.720962536	0.659	0.364
***Ms4a14***	1.98663 × 10^−8^	0.000348316	1.455458598	0.351	0.133
***Gabarap***	2.09845 × 10^−8^	0.000367921	0.568343096	0.892	0.679
***Tlr7***	2.23986 × 10^−8^	0.000392715	0.626407298	0.838	0.583
***Lpcat3***	2.31949 × 10^−8^	0.000406677	0.683349577	0.773	0.485
***Ap2m1***	2.50936 × 10^−8^	0.000439965	0.475606242	0.962	0.855
***Pqlc2***	2.58033 × 10^−8^	0.000452409	0.866455894	0.681	0.383
***Ric1***	2.61956 × 10^−8^	0.000459288	0.777490736	0.692	0.429
***Sbf2***	2.73846 × 10^−8^	0.000480134	1.302920849	0.346	0.123
***Atp6v1b2***	2.82003 × 10^−8^	0.000494436	0.435972398	0.962	0.824
***Msr1***	2.91675 × 10^−8^	0.000511394	0.492929478	0.984	0.738
***Kcnab2***	2.94156 × 10^−8^	0.000515744	0.739127343	0.735	0.46
***Fam50a***	3.39742 × 10^−8^	0.00059567	0.612767254	0.746	0.441
***Impdh1***	3.39933 × 10^−8^	0.000596005	0.918119702	0.373	0.139
***Tmem38b***	3.52918 × 10^−8^	0.000618772	0.917402016	0.6	0.315
***Itgav***	4.06522 × 10^−8^	0.000712755	0.527634198	0.897	0.781
***Fam219a***	4.15736 × 10^−8^	0.00072891	1.186725551	0.362	0.136
***Fmnl2***	4.16191 × 10^−8^	0.000729708	0.875835833	0.568	0.309
***Plekhm2***	4.27221 × 10^−8^	0.000749047	0.595665205	0.876	0.602
***Abhd5***	4.30258 × 10^−8^	0.000754372	1.222364316	0.4	0.17
***Xdh***	4.30983 × 10^−8^	0.000755642	0.509341687	0.93	0.679
***Sccpdh***	4.33801 × 10^−8^	0.000760583	1.360550787	0.265	0.077
***Mcm6***	4.53623 × 10^−8^	0.000795336	1.209238343	0.346	0.13
***Dync1i2***	4.54795 × 10^−8^	0.000797393	0.689194751	0.778	0.528
***Samd8***	4.78034 × 10^−8^	0.000838137	0.686218417	0.665	0.383
***Hyou1***	5.21002 × 10^−8^	0.000913473	0.508038185	0.946	0.719
***Prdx4***	5.22303 × 10^−8^	0.000915754	0.674518358	0.524	0.253
***Qsox2***	5.24954 × 10^−8^	0.000920401	1.120522986	0.292	0.093
***Tbc1d24***	5.29393 × 10^−8^	0.000928186	0.926275505	0.368	0.139
***Srm***	5.30484 × 10^−8^	0.000930097	1.087415671	0.351	0.13
***Als2***	5.33109 × 10^−8^	0.000934701	1.107768168	0.351	0.13
***Ldlrad3***	5.41124 × 10^−8^	0.000948753	0.709045581	0.395	0.16
***P4hb***	5.41493 × 10^−8^	0.0009494	0.453871357	0.968	0.818
***Pla2g15***	5.42727 × 10^−8^	0.000951563	0.628579296	0.876	0.593
***Psmd7***	5.53561 × 10^−8^	0.000970559	0.670682697	0.751	0.435
***Selenoi***	6.33318 × 10^−8^	0.001110397	0.909250324	0.378	0.148
***S100a8***	6.44518 × 10^−8^	0.001130034	0.828189525	0.27	0.083
***H6pd***	6.77639 × 10^−8^	0.001188105	0.680965895	0.757	0.494
***Cd109***	6.87945 × 10^−8^	0.001206175	3.041315858	0.151	0.025
***Vps50***	7.15323 × 10^−8^	0.001254176	0.816946523	0.524	0.244
***Tom1***	7.73419 × 10^−8^	0.001356035	0.658458934	0.595	0.296
***Ebpl***	7.93311 × 10^−8^	0.001390912	1.07456424	0.335	0.123
***Eif4a1***	8.06142 × 10^−8^	0.001413409	0.448984094	0.941	0.815
***Tmem126a***	8.5646 × 10^−8^	0.001501631	0.830655174	0.459	0.204
***Alg1***	9.27422 × 10^−8^	0.001626048	0.805848643	0.649	0.37
***Cyp4v3***	9.27432 × 10^−8^	0.001626066	0.810996989	0.622	0.315
***Kdelr2***	9.43259 × 10^−8^	0.001653816	0.614633401	0.8	0.509
***Mmp27***	9.45136 × 10^−8^	0.001657106	3.792036784	0.114	0.009
***Ccdc90b***	1.03987 × 10^−7^	0.00182321	1.308479445	0.276	0.09
***Calm1***	1.05123 × 10^−7^	0.001843119	0.472192433	0.962	0.877
***Ap5s1***	1.06427 × 10^−7^	0.001865989	1.118068629	0.378	0.157
***Ptgr1***	1.07877 × 10^−7^	0.001891402	1.353629763	0.319	0.117
***Itga9***	1.09916 × 10^−7^	0.001927156	1.236867554	0.286	0.102
***Ptpra***	1.10268 × 10^−7^	0.001933337	0.496559652	0.908	0.722
***Cacul1***	1.20586 × 10^−7^	0.002114236	0.772253387	0.719	0.444
***Ubl4a***	1.26532 × 10^−7^	0.002218488	0.744235632	0.497	0.231
***Plgrkt***	1.31045 × 10^−7^	0.002297604	0.487397892	0.73	0.457
***Gnptg***	1.42141 × 10^−7^	0.002492152	0.915167171	0.589	0.324
***Cad***	1.44525 × 10^−7^	0.002533965	0.916781697	0.362	0.145
***Cd5l***	1.4748 × 10^−7^	0.002585775	2.801895181	0.103	0.006
***Sox4***	1.48316 × 10^−7^	0.002600421	1.141646137	0.308	0.111
***Fkbp2***	1.48574 × 10^−7^	0.002604949	0.518205757	0.919	0.688
***Gdpd5***	1.60602 × 10^−7^	0.002815835	1.350517911	0.292	0.102
***Dag1***	1.64975 × 10^−7^	0.002892505	0.945170266	0.497	0.241
***Myo1e***	1.65733 × 10^−7^	0.00290579	0.758703102	0.562	0.296
***Pdgfa***	1.67141 × 10^−7^	0.002930476	0.975026744	0.438	0.194
***Cpt1a***	1.79665 × 10^−7^	0.003150059	0.504109074	0.854	0.602
***Chpt1***	1.80333 × 10^−7^	0.003161779	1.018785629	0.438	0.198
***Wdr91***	1.8783 × 10^−7^	0.003293217	0.994780045	0.551	0.321
***Cnot9***	1.88007 × 10^−7^	0.003296324	1.03820781	0.514	0.269
***Lgals3bp***	2.10375 × 10^−7^	0.003688513	0.610698195	0.811	0.586
***Galk2***	2.13643 × 10^−7^	0.003745798	0.792310719	0.589	0.324
***Rpn1***	2.15226 × 10^−7^	0.003773549	0.470174685	0.93	0.843
***Arl6ip1***	2.17796 × 10^−7^	0.003818613	0.559125751	0.892	0.698
***Vdac1***	2.22021 × 10^−7^	0.003892702	0.87051255	0.4	0.16
***Piezo1***	2.43871 × 10^−7^	0.004275784	0.464055195	0.93	0.676
***Odc1***	2.50094 × 10^−7^	0.00438489	0.714109814	0.519	0.262
***Stt3b***	2.53468 × 10^−7^	0.004444054	0.495058013	0.919	0.756
***Anxa7***	2.62813 × 10^−7^	0.004607894	0.643890851	0.778	0.58
***Gramd1b***	2.72519 × 10^−7^	0.004778068	0.764699511	0.616	0.349
***Tbcb***	2.83323 × 10^−7^	0.004967507	0.624832343	0.751	0.534
***Rexo2***	2.89361 × 10^−7^	0.005073374	0.597823437	0.714	0.42
***Pno1***	2.93384 × 10^−7^	0.005143906	0.927749872	0.503	0.259
***Tpi1***	3.23869 × 10^−7^	0.0056784	0.7594751	0.568	0.296
***Mcoln2***	3.30414 × 10^−7^	0.005793141	1.729789689	0.173	0.037
***Noc2l***	3.49948 × 10^−7^	0.006135635	0.638147124	0.757	0.488
***Nudt9***	3.65409 × 10^−7^	0.006406719	0.728004671	0.568	0.315
***Sema4b***	3.73698 × 10^−7^	0.006552045	0.750243724	0.47	0.219
***Kpna4***	4.19224 × 10^−7^	0.007350247	0.612433382	0.849	0.648
***Pigk***	4.21041 × 10^−7^	0.007382113	0.674630651	0.643	0.401
***Tbxas1***	4.21245 × 10^−7^	0.007385684	0.579308301	0.827	0.596
***Nop2***	4.21704 × 10^−7^	0.007393728	0.825035469	0.503	0.244
***Pcna***	4.25487 × 10^−7^	0.007460058	0.637977303	0.827	0.596
***Psat1***	4.27762 × 10^−7^	0.00749995	1.523880027	0.2	0.052
***Ranbp1***	4.34143 × 10^−7^	0.007611836	0.746341137	0.632	0.38
***Tmx4***	4.5295 × 10^−7^	0.007941576	0.53830866	0.627	0.343
***Ssh3***	4.61642 × 10^−7^	0.008093975	0.707468612	0.654	0.364
***Fdx2***	4.77175 × 10^−7^	0.008366303	0.709620663	0.459	0.225
***C2cd2***	4.81831 × 10^−7^	0.008447935	1.261326572	0.265	0.09
***Tuba1b***	4.90281 × 10^−7^	0.0085961	0.509368964	0.784	0.543
***Tcirg1***	4.95472 × 10^−7^	0.008687106	0.33538394	0.989	0.954
***Spryd7***	5.021 × 10^−7^	0.008803312	1.003319536	0.346	0.136
***Uchl5***	5.27935 × 10^−7^	0.009256282	0.712586956	0.535	0.284
***Qtrt1***	5.3051 × 10^−7^	0.009301426	1.378645253	0.297	0.111
***Sh3glb2***	5.32603 × 10^−7^	0.009338123	0.951974138	0.4	0.173
***Zfp469***	5.33015 × 10^−7^	0.009345357	1.695696197	0.086	0.003
***Abcd3***	5.71729 × 10^−7^	0.01002413	0.976532697	0.346	0.136
***Ddost***	5.74333 × 10^−7^	0.01006978	0.38697453	0.951	0.855
***Tmed9***	5.7902 × 10^−7^	0.010151962	0.436973293	0.957	0.852
***Gas2l1***	5.93707 × 10^−7^	0.01040947	0.678144503	0.514	0.259
***Cd300lf***	6.01582 × 10^−7^	0.010547534	0.438173514	0.919	0.722
***Cd37***	6.04992 × 10^−7^	0.010607317	0.470878387	0.784	0.515
***Vps13c***	6.47534 × 10^−7^	0.011353212	0.52976155	0.886	0.642
***Nasp***	6.61976 × 10^−7^	0.011606432	0.995853998	0.47	0.235
***Ddhd1***	6.81638 × 10^−7^	0.011951159	0.806299389	0.643	0.383
***Gpc1***	6.98229 × 10^−7^	0.012242051	0.80427869	0.422	0.201
***Cd99l2***	7.21958 × 10^−7^	0.012658088	1.234305347	0.2	0.052
***St13***	7.45275 × 10^−7^	0.01306691	0.684981408	0.622	0.336
***Pdlim4***	7.62701 × 10^−7^	0.013372441	1.032776197	0.357	0.151
***Slc36a1***	7.9702 × 10^−7^	0.013974155	0.52066872	0.822	0.586
***Irs2***	8.04369 × 10^−7^	0.01410301	0.68851624	0.627	0.358
***Tmem106c***	8.32456 × 10^−7^	0.014595457	1.147691685	0.259	0.09
***Sumf2***	8.33639 × 10^−7^	0.014616197	1.011227467	0.378	0.164
***Stom***	8.40217 × 10^−7^	0.014731519	0.649984611	0.692	0.401
***Tsfm***	8.75575 × 10^−7^	0.015351452	0.83225706	0.351	0.142
***Cotl1***	8.83996 × 10^−7^	0.015499099	0.322295423	0.989	0.969
***Arl11***	8.85817 × 10^−7^	0.015531028	0.587045656	0.757	0.497
***Plpp5***	8.91886 × 10^−7^	0.015637429	0.762012733	0.454	0.225
***Il18bp***	8.99307 × 10^−7^	0.015767551	1.440828923	0.276	0.102
***Mif***	9.02623 × 10^−7^	0.015825681	0.953428763	0.557	0.315
***Reps2***	9.41549 × 10^−7^	0.016508171	1.111866726	0.2	0.056
***Rhbdd3***	9.98324 × 10^−7^	0.017503615	1.470623443	0.249	0.083
***Selenon***	1.03209 × 10^−6^	0.018095548	0.62639376	0.584	0.315
***Asna1***	1.08422 × 10^−6^	0.019009664	0.617004004	0.746	0.481
***Smyd3***	1.0846 × 10^−6^	0.019016232	0.835213344	0.303	0.117
***Tnf***	1.08984 × 10^−6^	0.019108089	0.56710465	0.373	0.16
***Igf1***	1.14934 × 10^−6^	0.020151446	2.092150868	0.13	0.022
***P3h1***	1.18193 × 10^−6^	0.020722788	1.373711045	0.189	0.049
***Slc12a7***	1.31648 × 10^−6^	0.023081931	0.649481676	0.562	0.312
***Dera***	1.31939 × 10^−6^	0.023132907	0.702999698	0.416	0.191
***Htatip2***	1.47953 × 10^−6^	0.025940513	0.6633332	0.605	0.327
***Commd10***	1.49148 × 10^−6^	0.026150185	0.828342936	0.449	0.219
***Nacc2***	1.55288 × 10^−6^	0.027226687	0.718002419	0.373	0.157
***Rngtt***	1.59732 × 10^−6^	0.028005899	0.836477835	0.368	0.157
***Memo1***	1.61871 × 10^−6^	0.028380759	0.643397469	0.616	0.34
***Dtx4***	1.62297 × 10^−6^	0.028455572	0.515879884	0.843	0.577
***Chst14***	1.63821 × 10^−6^	0.028722676	0.678012685	0.541	0.281
***Atp6v1d***	1.63888 × 10^−6^	0.02873454	0.484808718	0.849	0.608
***Cnih1***	1.68569 × 10^−6^	0.029555141	0.526473285	0.714	0.463
***Clpp***	1.70997 × 10^−6^	0.029980909	0.81101501	0.4	0.179
***Cdkn2aipnl***	1.72032 × 10^−6^	0.030162313	0.641860756	0.368	0.154
***Sqstm1***	1.7747 × 10^−6^	0.03111578	0.761923427	0.697	0.463
***Serac1***	1.77537 × 10^−6^	0.031127533	1.086861366	0.249	0.086
***Ide***	1.82236 × 10^−6^	0.031951372	0.663405236	0.632	0.373
***Cers2***	1.83561 × 10^−6^	0.03218368	0.434433796	0.924	0.79
***Insl6***	1.91792 × 10^−6^	0.033626918	1.955338154	0.141	0.028
***Ezr***	2.2321 × 10^−6^	0.039135378	0.548224017	0.762	0.503
***Ctnnd1***	2.28475 × 10^−6^	0.040058494	0.607419269	0.649	0.373
***Scpep1***	2.39404 × 10^−6^	0.041974747	0.384523304	0.962	0.883
***Bst1***	2.39582 × 10^−6^	0.042005863	0.498080154	0.881	0.617
***Tubb2a***	2.42234 × 10^−6^	0.042470841	0.817658292	0.573	0.327
***Trio***	2.44727 × 10^−6^	0.042908052	0.652566787	0.681	0.444
***Trim37***	2.45645 × 10^−6^	0.043068879	0.883741079	0.297	0.114
***Ptrh1***	2.49254 × 10^−6^	0.043701629	1.477436029	0.2	0.059
***Nrg4***	2.49926 × 10^−6^	0.043819552	3.095833937	0.103	0.012
***Slc11a2***	2.53024 × 10^−6^	0.044362624	0.853062036	0.459	0.244
***Iqgap2***	2.54143 × 10^−6^	0.044558852	0.523891163	0.73	0.457
***Eef1e1***	2.62347 × 10^−6^	0.045997299	1.26106159	0.319	0.133
***Pcdhgb4***	2.67228 × 10^−6^	0.046853107	3.122928618	0.086	0.006
***Psmc1***	2.70695 × 10^−6^	0.047461019	0.59830703	0.751	0.485
***Ube2q2***	2.73025 × 10^−6^	0.047869554	0.8052921	0.557	0.324
***Tbc1d2***	2.77264 × 10^−6^	0.048612636	0.950332669	0.276	0.105
***Snx30***	2.77852 × 10^−6^	0.048715724	0.703317436	0.535	0.281
